# 1,3,5-Triazine as Branching Connector for the Construction of Novel Antimicrobial Peptide Dendrimers: Synthesis and Biological Characterization

**DOI:** 10.3390/ijms25115883

**Published:** 2024-05-28

**Authors:** Rotimi Sheyi, Jessica T. Mhlongo, Marta Jorba, Ester Fusté, Anamika Sharma, Miguel Viñas, Fernando Albericio, Paula Espinal, Beatriz G. de la Torre

**Affiliations:** 1 Peptide Science Laboratory, School of Chemistry and Physics, University of KwaZulu-Natal, Durban 4001, KwaZulu-Natal, South Africa; ebenrotex4fun@gmail.com (R.S.); jtmhlongo91@gmail.com (J.T.M.); anamika.aug14@gmail.com (A.S.); albericio@ukzn.ac.za (F.A.); 2 School of Laboratory Medicine and Medical Sciences, College of Health Sciences, University of KwaZulu-Natal, Durban 4041, KwaZulu-Natal, South Africa; 3 Laboratory of Molecular Microbiology & Antimicrobials, Department of Pathology and Experimental Therapeutics, Faculty of Medicine & Health Sciences, IDIBELL—University of Barcelona, Campus Bellvitge, 08907 L’Hospitalet de Llobregat, Barcelona, Spain; m.jorba.pedrosa@gmail.com (M.J.); esterfustedominguez@ub.edu (E.F.); mvinyas@ub.edu (M.V.); 4 Department of Public Health, Mental Health and Maternal and Child Health Nursing, University of Barcelona, Campus Bellvitge, 08907 L’Hospitalet de Llobregat, Barcelona, Spain; 5 Networking Centre on Bioengineering, Biomaterials and Nanomedicine (CIBER-BBN), Department of Organic Chemistry, University of Barcelona, Martí i Franqués 1-11, 08028 Barcelona, Barcelona, Spain

**Keywords:** antimicrobials, efflux pump inhibitors, multidrug-resistant bacteria (MDR), peptide dendrimers, solid-phase peptide synthesis (SPPS), 2,4,6-Trichloro-1,3,5-triazine (TCT)

## Abstract

Peptides displaying antimicrobial properties are being regarded as useful tools to evade and combat antimicrobial resistance, a major public health challenge. Here we have addressed dendrimers, attractive molecules in pharmaceutical innovation and development displaying broad biological activity. Triazine-based dendrimers were fully synthesized in the solid phase, and their antimicrobial activity and some insights into their mechanisms of action were explored. Triazine is present in a large number of compounds with highly diverse biological targets with broad biological activities and could be an excellent branching unit to accommodate peptides. Our results show that the novel peptide dendrimers synthesized have remarkable antimicrobial activity against Gram-negative bacteria (*E. coli* and *P. aeruginosa*) and suggest that they may be useful in neutralizing the effect of efflux machinery on resistance.

## 1. Introduction

The emergence of multidrug-resistant (MDR) bacteria coupled with the lack of new effective antimicrobials is a major public health issue worldwide [1,2]. Thus, the search for new strategies to fight against infections caused by MDR bacteria is a clear demand not only for the pharmaceutical industry, but also for the entire society. Antimicrobial peptides (AMPs) have become an alternative to conventional small molecules-based antimicrobial drugs [3]. The main reason is that these peptides are present in all living organisms as part of the defense of innate immunity [4]. The great potential of AMPs as antimicrobial drugs lies mainly in their broad-spectrum activity and low-resistance induction [5]. Additionally, they have shown synergistic effects when they are tested together with conventional antibiotics and modulating effects on the immune system [6]. Nevertheless, AMPs have several drawbacks that should be overcome, such as their short half-life because of the degradation by proteases, hemolytic activity or toxicity to the host.

To minimize these problems, different topological peptide constructions have been proposed, mainly based on cyclic structures [7,8,9]. However, the introduction of a cycle increases their synthetic complexity, synthesis of a protected peptide and further cyclization. Another alternative that has received great interest from a therapeutic point of view not only regarding the development of antimicrobial drugs is the construction of dendritic or branched structures [10,11,12,13,14]. Dendrimers, which can be defined as repetitive chemical moieties organized around branching units following a tree-like structure [15], exert broad biological activity and are thus highly attractive for pharmaceutical innovation and development. In general, peptide-based dendrimers show more resistance to the degradation caused by proteases due probably to the dendrimeric peptides’ spatial disposition that gives a kind of steric protection. Most importantly, the dendrimeric peptides show better activity when compared to the corresponding peptide monomers. This can also be interpreted due to a higher concentration of active species in situ [16].

Having said that, the construction of novel chemical platforms for holding AMPs is of great interest. 1,3,5-Triazine is an intriguing molecule with a broad application in several industrial sectors such as the pharmaceutical and agrochemical sectors, and the one developed around new materials [17]. The synthetic precursor of the 1,3,5-triazine scaffold is 2,4,6-trichloro-1,3,5-triazine (TCT). The three reactive Cl atoms can sequentially and independently be substituted by different N, O, S containing nucleophiles exploiting the orthogonal chemoselectivity of the TCT to render very diverse compounds. Importantly and from a biological perspective, the 1,3,5-triazine could be defined as a promiscuous scaffold because there are a large number of compounds build up around the triazine that show interesting biological activities such as anti-inflammatory, anti-mycobacterial, anti-viral, anti-cancer, etc. [18]. The singular structural characteristics of 1,3,5-triazine make it an interesting moiety. It presents a planar hexagonal structure, and when positions 2,4,6 are substituted with nucleophiles such as amine groups, the triazine core resembles the biological purine and pyrimidine heterocycles.

**Figure 1:**
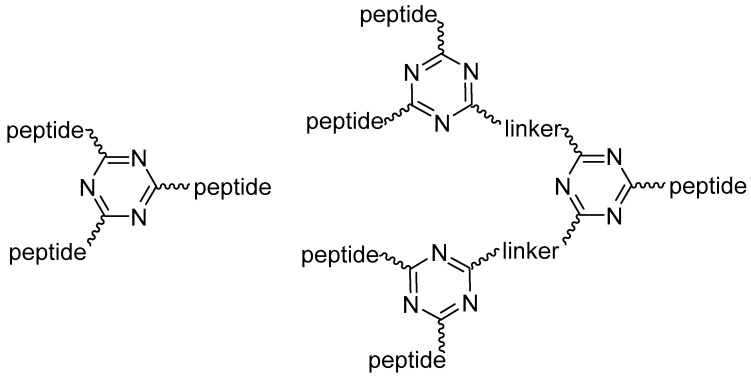
Generation 1 and 2 (G1, G2) of triazine-based dendrimeric peptides.

Thus, 1,3,5-triazine could provide an excellent branching unit to accommodate peptides. Figure 1 shows generations 1 and 2 (G1, G2) of the triazine-based dendrimeric peptides used in this work.

As peripheral moieties of our dendrimers, and based on previous reports [19,20,21,22], we chose a short peptide based on the repetitive alternance of two residues, one hydrophobic, Leu, and one cationic, Lys. Peptides based on this kind of sequences have been previously studied as antimicrobial agents and were found to be interesting because of their ability to form β-stranded structures when they bound to membranes that can result in oligomeric β-sheets or long amyloid-like cross-β-sheet fibrils. Moreover, in general, the hemolysis shown by this kind of compound is low [23,24]. Thus, H-Leu-Lys-Leu-Lys-Leu-Lys-NH_2_ [H-(LK)_3_-NH_2_] (**A**) was chosen as the number of Leu-Lys repetitions to be installed in each branch. Herein, we explored the use of 1,3,5-triazine on SPPS to build up dendrimeric peptides and study their antimicrobial activity as well as gain some insights into their mechanisms of action.

## 2. Results and Discussion

### 2.1. Synthetic Strategies for Dendrimer-Based Peptides

Our strategy was to synthesize peptide dendrimers completely in the solid phase, starting with a peptide, followed by the introduction of the 1,3,5-triazine via TCT reaction by replacing just one Cl. Next, the two remaining Cl atoms were substituted by an amine function, which in turn allowed the simultaneous stepwise elongation of two peptide chains to give dendrimer G1. For the next generation of the dendrimer (G2), the same strategy was repeated.

For the efficient application of this scheme, two factors should be considered. First, the synthesis of these macromolecules is not straightforward because the introduction of each branching unit doubles the capacity of the resin, favors interchain interaction and jeopardizes the continuous growth of the peptide chain. In other words, the branching unit brings about “congestion” of the resin. Second, when 1,3,5-triazine is used as a branching unit, it is important to control the three substitutions on its TCT precursor as the first substitution is usually performed at 0 °C, the second at room temperature (rt) and the third at >50 °C [25]

Our group has previously demonstrated that amines and phenols have the best chemical functions for reacting with chloro-triazine derivatives [26,27,28]. The introduction of a phenol on the first position of the triazine core has the additional advantage of enhancing the reactivity of the remaining two Cl reactive points of the triazine core towards nucleophiles as a result of the electron-withdrawing effect of this moiety, thereby allowing the reaction to take place under mild temperatures [27,28]

**Scheme 1:**
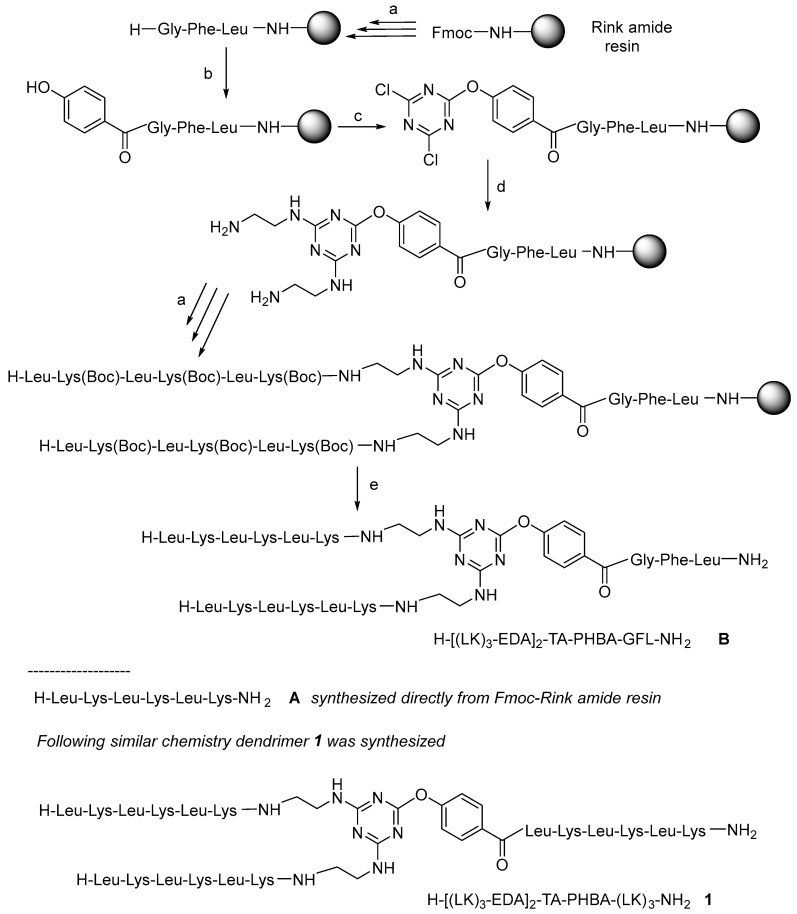
General protocol used in the synthesis of dendrimeric constructs: (a) Solid-phase peptide synthesis (SPPS) at rt: (i) Fmoc removal: 20% *v/v* piperidine in DMF, (1 × 1 min + 1 × 7 min), resin washing: DMF (3×), (ii) Fmoc-AA-OH (3 eq), DIC (3 eq), OxymaPure (3 eq) (60 min); resin washing: DMF (3×) (ii); (b) (i) p-hydroxybenzoic acid (3 eq), DIC (3 eq), OxymaPure (3 eq), double coupling; (ii) 20% *v/v* piperidine in DMF, (1 × 1 min + 1 × 7 min), resin washing: DMF (3×); (c) (i) 2,4,6-trichloro-1,3,5-triazine (3 eq), DIEA (3 eq) in DCM at −20 °C, 1 h; (d) EDA (50 eq), DIEA (50 eq) at rt overnight; (e) TFA-TIS-H_2_O (95:2.5:2.5), at r.t, 1 h, and RP-HPLC purification.

All the peptides in this study were synthesized as C-terminal amides using a fluorenylmethoxycarbonyl (Fmoc)/tert-butyl (tBu) protection scheme on a Fmoc-RinkAmide-resin and N,N’-diisopropylcarbodiimde (DIC)/2-cyano-2-(hydroxyimino)acetate (OxymaPure) as the coupling method [29]. To test the feasibility of our proposal, first of all, the tripeptide (Gly-Phe-Leu) was first incorporated to separate the resin from the growing molecule. Next, the *p*-hydroxybenzoic acid (PHBA) was incorporated directly into the N-terminal of the Gly residue without phenol protection which could lead to double or even triple incorporation of the PHBA unit. To prevent the possible extra attached PHBA units, the peptidyl-resin was treated with a solution of 20% *v/v* piperidine in DMF (the same solution as that used to remove the Fmoc group) to hydrolyze the possible phenyl ester formed. After checking the successful incorporation of a single PHBA moiety, the triazine was introduced in the presence of DIEA at −20 °C for 1 h. Ethylenediamine (EDA) was then added in excess in the presence of DIEA at rt for 12 h. Finally, two copies of [Leu-Lys(Boc)]_3_ were built up on the construct to render a first-generation (G1) dendrimer H-[(LK)_3_-EDA]_2_-TA-PHBA-GFL-NH_2_ (**B**). Scheme 1 shows the conditions used for the synthesis of **B**.

After verifying that **B** showed preliminary antimicrobial activity, although the hexapeptide **A** and (Ac-EDA)_2_-TA-PHBA-GFL-NH_2_ (**C**) were completely inactive (Table 1) (**B** can be interpreted as a conjugation of **A** and **C**), the G1 and G2 peptide dendrimers were prepared as shown in Scheme 1, where the linker peptide Gly-Phe-Leu was substituted by the hexapeptide (Leu-Lys)_3_ already present in **B**. The synthesis of G1 (**1**) (Scheme 1) took place as expected. However, during the synthesis of G2 (not shown in the scheme), we were aware of a major problem in analyzing the byproducts obtained. During the EDA reaction with the dichloro-triazine resins, the EDA was not only substituting the Cl, but also the PHBA with concomitant cleavage of the triazine moiety from the growing dendrimer and the formation of truncated dendrimers (Figure S1). This observation is explained by the fact that the phenol of the PHBA moiety is also a good leaving group.

**Scheme 2:**
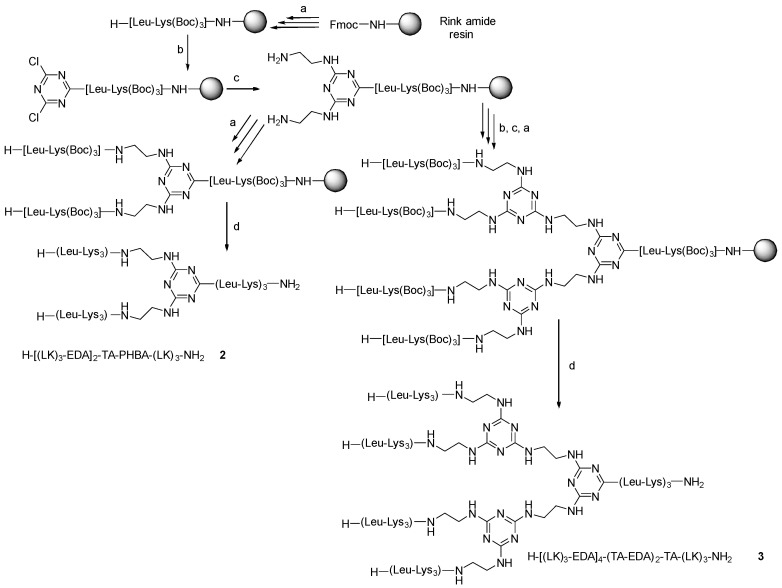
Protocol used in the synthesis of dendrimer **2** and **3**. (a) Solid-phase peptide synthesis (SPPS) at rt: (i) Fmoc removal: 20% *v/v* piperidine in DMF, (1 × 1 min + 1 × 7 min), resin washing: DMF (3×), (ii) Fmoc-AA-OH (3 eq), DIC (3 eq), OxymaPure (3 eq) (60 min); resin washing: DMF (3×) (b) (i) 2,4,6-trichloro-1,3,5-triazine (3 eq), DIEA (3 eq) in DCM at −20 °C, 1 h; (c) EDA (50 eq), DIEA (50 eq) at 45 °C for 3 h; (d) TFA-TIS-H_2_O (95:2.5:2.5), at r.t, 1 h, and RP-HPLC purification.

Thus, the synthesis of G1 and G2 was repeated, but without the incorporation of PHBA. In this case, without the PHBA as an enhancer, the first incorporation of TCT was carried out at rt for 1 h, while the replacement of the two remaining Cl by EDA was performed at 45 °C for 3 h. Two (H-[(LK)_3_-EDA]_2_-TA-(LK)_3_-NH_2_, (**2**) and four (H-[(LK)_3_-EDA]4-(TA-EDA)_2_-TA-(LK)_3_-NH_2_, (**3**) peptide dendrimers were obtained (Scheme 2).

### 2.2. Minimum Inhibitory Concentrations (MIC)

As a preliminary evaluation of antimicrobial activity, MICs were determined against Gram-positive and Gram-negative bacteria (Table 1). As mentioned before, in addition to dendrimers **1**, **2** and **3,** the hexapeptide **A** and the moiety consisting of a copy of the peptide **A** linked to the branching moiety, i.e., (Ac-EDA)_2_-TA-PHBA-(LK)_3_-NH_2_ (**D**) and (Ac-EDA)_2_-TA-(LK)_3_-NH_2_ (**E**), were also tested. The hexapeptide H-(Leu-Lys)_3_-NH_2_ (**A**) and the three acetylated cores (**C**, **D**, **E**) did not show any remarkable activity against any of the strains tested (Table 1). Significant activity was found only for the full dendrimers, meaning **1**, **2** and **3**. Interestingly, the dendrimer with the three copies of (Leu-Lys)_3_ (**1**) shows higher activity than its analog with two copies of (Leu-Lys)_3_ and the linker GFL (**B**). This observation indicates the importance of multiple copies of (Leu-Lys)_3_. Comparing the activities of **1** and **2,** which differ in the PHBA moiety, allowed us to conclude that the activity in the presence of PHBA is not advantageous and that the use of this linker leads to many synthetic problems.

Moreover, moving from G1 (**2**) to G2 (**3**) did not enhance antimicrobial activity. On the basis of these results, it could be concluded that the greater synthetic effort required to reach a G2 triazine branched dendrimer is not worthwhile.

Finally, we studied the relevance of the topology of the molecule. To this end, G1 (**2**) was compared with a similar G1 dendrimer using Lys as a branching unit (**E**) and with the lineal peptide H-(Leu-Lys)_9_-NH_2_ (**G**), which holds the same number of Leu-Lys units as the two G1 dendrimers (**2**, **F**). Interestingly, the dendrimer with Lys as a branching point was much less active than the triazine-based dendrimer. This observation indicates that although the triazine moiety does not exhibit antimicrobial activity per se, its presence in the branched peptide plays a significant role. Dendrimers **1**, **2** and **3** showed greater activity against Gram-negative bacteria and were therefore selected for further analysis of antimicrobial activity and mechanism of action. *S. aureus* was not included due to the lack of activity of the dendrimers against the reference tested strain.

It is noteworthy that none of these dendrimers were hemolytic at concentrations up to 1 mg/mL. In addition, the cytotoxicity of the compounds on L-929 and HepG2 cells was almost negligible at concentrations as high as 64 µg/mL.

### 2.3. Antimicrobial Activity in MDR Isolates

Although in principle, peptides and antibiotics do not share resistance mechanisms, it seemed convenient to explore the activity of the synthesized molecules on multidrug-resistant (MDR) clinical isolates. Dendrimers 1 and 2 were tested against a total of six MDR clinical isolates of *E. coli* and five MDR clinical isolates of *P. aeruginosa* (Table 2). The MICs of dendrimer **1** in four of the MDR *E. coli* isolates were identical to the one determined in the ATCC counterpart (8 µg/mL), while in the other two, the MICs were higher, reaching a value of 32 µg/mL. In the MDR *P. aeruginosa* strains, only one showed the same value as the ATCC counterpart (16 µg/mL) and the other four resulted in higher MICs. Regarding dendrimer **2**, the MIC values in MDR *E. coli* varied between 2 and 8 µg/mL. In the MDR *P. aeruginosa* group, the values in all strains tested were between 16 and 32 µg/mL, similar to the MIC value in the ATCC strain (16 µg/mL). These results show that susceptibilities to dendrimers are almost identical in susceptible and MDR isolates. In some cases such as *E. coli* 208691 and *P. aeruginosa* P15 and others, dendrimer 2 displays much more favorable values than the controls.

The most common approach used to establish the antimicrobial capacity of antibiotics, disinfectants and other agents is by determining the minimum inhibitory concentration (MIC) following standardized procedures recommended by U.S. or European agencies. This parameter gives information only on the culture state after the period of incubation in standard conditions (18 h or more) [30]. The antibacterial effect may also be demonstrated in dynamic experiments in which the effect of antimicrobials on the growth parameters and/or the kinetics of bacterial killing are studied. These effects are much more illustrative because they allow the tracking of the entire process and not just the start and end points. Furthermore, to explore the mechanism of antimicrobial action, various approaches can be adopted, such as examining the effect of compounds on membranes, biofilms, efflux machinery and others.

### 2.4. Effect on Bacterial Growth

**Figure 2:**
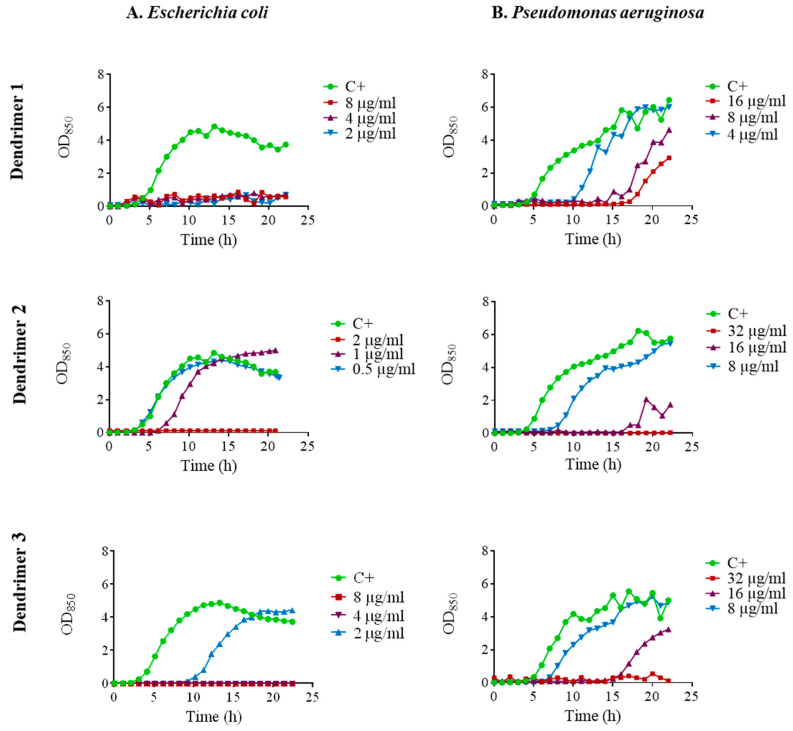
Growth curves of (**A**). *E. coli* ATCC 25922; (**B**). *P. aeruginosa* ATCC 27853 in the presence of dendrimers **1**, **2** and **3** at MIC (red line), ½ MIC (purple line) and ¼ MIC (blue line). Control without peptide (green line).

The kinetics of antimicrobial activity were assessed by plotting growth curves in the presence of the three selected peptide dendrimers (**1**, **2** and **3**). We tested the MIC, ½ MIC and ¼ MIC. Dendrimer **1** exhibited remarkable antimicrobial activity against *E. coli* ATCC 25922 since it fully inhibited growth at the three concentrations tested (Figure 2A). In contrast, dendrimer **2** achieved full growth inhibition only at the MIC, while dendrimer **3** inhibited the growth at the MIC and ½ MIC and caused a 6 h prolongation of lag phase at ¼ MIC.

In *P. aeruginosa* ATCC 27853, there was a noticeable growth delay in the presence of dendrimer **1** which was dependent on the concentration. In the case of dendrimers **2** and **3**, complete growth inhibition occurred at the MIC. When ½ MIC was applied, a marked delay in the growth was observed, and even some delay was observed at ¼ MIC (Figure 2B).

When considering the bacterial susceptibility to the compounds determined in *E. coli,* an apparent discrepancy was observed between the susceptibility values obtained by MIC and the growth curves. Using the microdilution method, the values were relatively high; for example, for dendrimer **1**, the MIC by microdilution was 8 µg/mL (Table 1). However, the MIC value deduced from microfermentor experiments was much lower (<2 µg/mL), as a concentration of 2 µg/mL was sufficient to completely inhibit detectable growth for 24 h. The reasons for this apparent contradiction have been addressed masterfully in a recent publication by Berryhill et al. [30]. In this work, it has been pointed out that although MIC is a useful parameter in pharmacodynamics when a comparison of antimicrobial susceptibilities of different isolates of the same species under identical in vitro conditions is needed, it has many limitations and cannot be the sole parameter for studying new antimicrobials. For example, the MIC does not take into account the influence of sub-inhibitory concentrations of the molecule and it does not provide information on the average growth rate, nor does it indicate the post-antimicrobial effect, among others. In fact, the MIC has been largely posed in question in research but the contribution mentioned [30] enlarged the criticism as it concludes that the mentioned limitations question the use of MIC not only in research but also as the unique pharmacodynamic parameter to develop therapeutically oriented protocols.

Similarly, dendrimer **3** completely inhibited growth at 4 µg/mL (½ MIC) and produced a delay of 10 h in the onset of detectable turbidity at lower concentrations. The discrepancy between the approaches is an interesting methodological paradox. In principle, the MIC, as determined by the CLSI or EUCAST standardized methods, is universally accepted for determining antimicrobial susceptibility. This parameter is useful in routine clinical practice; nevertheless, as stated earlier, it is a weak parameter in a research setting. Despite the high MIC values initially found in the search for new antimicrobials, further experiments commonly provide evidence of the efficacy of such products when tested using other techniques. In other words, when searching for new antimicrobials, high MIC values as determined by microdilution do not exclude a good antimicrobial effect under certain conditions and further experiments should be conducted. In addition, the possible occurrence of synergisms, inhibition of efflux machinery, etc., should be also explored.

### 2.5. Time-Kill Curves

**Figure 3:**
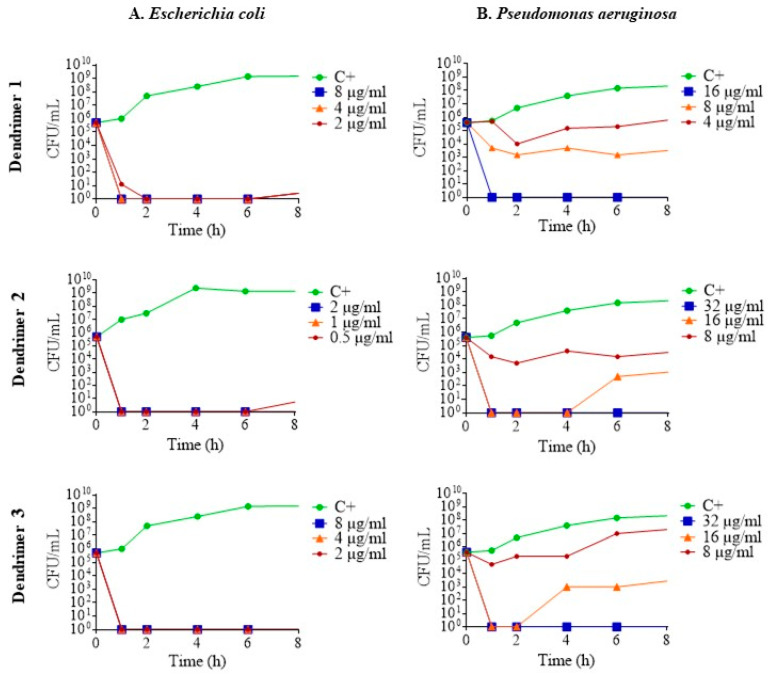
Time-kill curves of (**A**)*. E. coli* ATCC 25922; (**B**). *P. aeruginosa* ATCC 27853 in the presence of dendrimers 1, 2 and 3 at MIC (blue line), ½ MIC (orange line) and ¼ MIC (red line). Control without peptide (green line).

To determine the pharmacodynamics of the dendrimers, their death kinetics were examined by plotting time-kill curves. Regarding the results in *E. coli* ATCC 25922, at sub-inhibitory concentrations, the dendrimers killed all the bacteria in less than 1 h; that is to say, they act as a bactericide. After 6 h, eventually, some re-growth occurred, demonstrating a certain degree of bacterial survival (Figure 3A). In the case of *P. aeruginosa* ATCC 27853, the three dendrimers killed bacteria in a concentration-dependent manner. At MIC, complete eradication of bacteria was achieved in less than 1 h for all three dendrimers. Furthermore, at ½ MIC, dendrimers 2 and 3 showed higher antimicrobial activity than dendrimer 1 (Figure 3B).

The results of the antimicrobial activity and the kinetics through growth and death curves indicate that the peptide dendrimers **1**, **2** and **3** have remarkable activity against Gram-negative bacteria (*E. coli* and *P. aeruginosa)*.

### 2.6. Exploring the Mechanisms of Action

**Figure 4:**
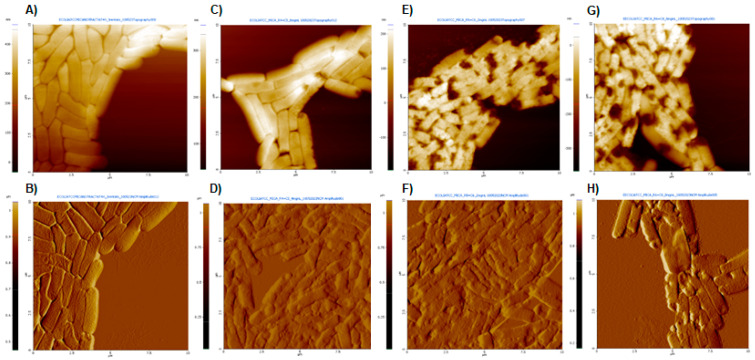
AFM images obtained at a scan size of 100 μm^2^. (**A**,**C**,**E**,**G**) Topography images and (**B**,**D**,**F**,**H**) amplitude images. (**A**,**B**) Untreated *E. coli;* (**C**,**D**) *E. coli* after treatment with dendrimer 1 at MIC (8 µg/mL); (**E**,**F**) *E. coli* after treatment with dendrimer 2 at MIC (2 µg/mL); (**G**,**H**) *E. coli* after treatment with dendrimer 3 at MIC (8 µg/mL).

AFM images of *E. coli* 25922 after treatment with dendrimers **1**, **2** and **3** at MIC, as well as untreated bacteria, are shown in Figure 4. The topography images (A, C, E, G) provide information on structural and surface differences, while the amplitude images show finer details of the alterations caused by the dendrimer treatments (B, D, F, H).

Untreated bacteria appeared as typical rod-shaped individuals with no damage to their outer membranes (Figure 4A). In contrast, significant surface damages were observed in the presence of peptide dendrimers **1**, **2** and **3**, with severely altered bacterial cell surfaces and the noticeable presence of some extracellular material (possibly debris) (Figure 4D,F,H). The surface disruption effects were similar for all three dendrimers. Similar results were observed in *E. coli* treated with peptides BP100 and pepR [31].

The eventual effect of the interaction between studied peptide dendrimers and artificial lipid bilayers was investigated by measuring the conductance of black lipid bilayers in the presence of the compounds. None of the dendrimers had the ability to induce measurable channels in planar lipid bilayers, since no noticeable increases in conductance were registered despite the wide range of both peptide concentrations and voltages applied across the bilayers during the experiments (−80, −120 and −180 mV). Thus, despite the marked disturbances induced by the dendrimers in the bacterial surface (shown in the AFM images), it seems unlikely that the effect occurs through altered permeability. The lack of channel formation could be because the interaction with the membranes will not result in the formation of channels, or it could be a consequence of the molecules studied requiring some protein component of the membrane itself that obviously the artificial bilayers do not contain. In any case, AFM imaging demonstrates significant disturbances of the bacterial surface upon incubation with the dendrimers, with a noticeable presence of some extracellular material (possibly leakage of cytoplasmic or periplasmic content).

Many cases of antibiotic resistance are due, at least in part, to the active efflux of antibiotic molecules by so-called efflux pumps. The search for molecules that inhibit the efflux machinery (efflux pump inhibitors, EPIs) is another major goal of microbiological research. As the ability to discover new antibiotics is seriously limited by costs and the interest of the pharmaceutical industry, the recovery of old antimicrobials is a field in full expansion, but this recovery requires adjuvants or chemosensitizers that help to overcome the resistance mechanisms. It has been shown that numerous discrepancies in the final restored susceptibility strongly depend on the antibiotic and the EPI studied [32].

**Figure 5:**
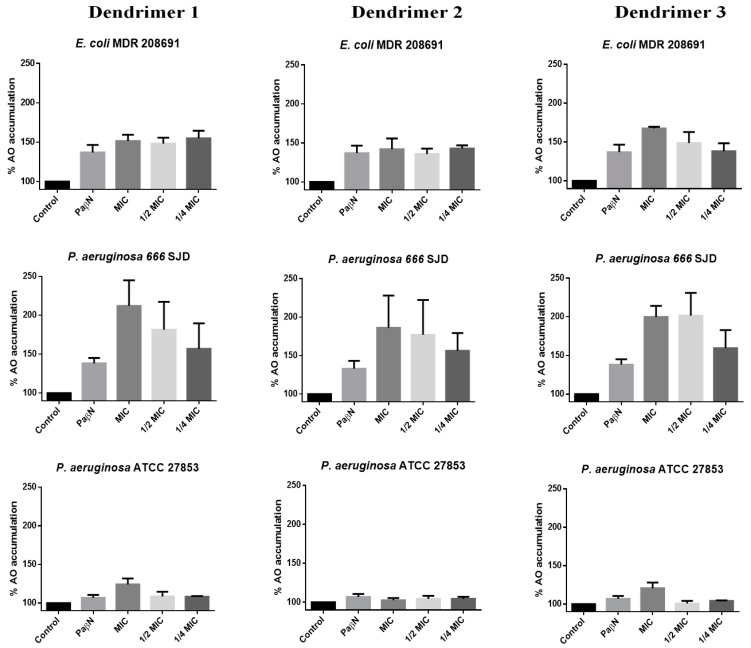
Percentage of Acridine Orange (AO) accumulation with dendrimers 1, 2 and 3. The control represents the assay with bacteria and AO. Assays were performed in *E. coli* 208691, *P. aeruginosa* 666 SJD and *P. aeruginosa* ATCC 27853.

Acridine orange (AO) is a good substrate for many efflux pumps such as acrAB in *E. coli* or Mex AB-OprM in *Pseudomonas* spp. [33,34]; thus, intracellular accumulation of AO strongly depends on the inhibition of the bacterial efflux pumps. We have measured the changes in AO uptake and accumulation in the presence of the three peptide dendrimers (Figure 5).

To confirm whether AO or dendrimers used in the experiments could affect the viability of the bacterial cells, confocal laser scanning microscopy (CLSM) assays were performed at 30 min of incubation. The results showed that the AO, at the concentrations used, did not affect the viability of the bacterial cells or the dendrimers in the period in which accumulation was measured (Figure S23).

The accumulation of AO by untreated bacteria (control) was considered 100%. In the presence of the EPI PaβN, AO accumulation increased by at least up to 140% in the clinical isolates included in the assays (*E. coli* 208691 and *P. aeruginosa* 666 SJD), while changes in AO accumulation by susceptible strains were almost negligible, as presumably, the efflux machinery has poor or no activity in these bacteria. In fact, the irrelevance of the efflux mechanisms in susceptible bacteria is to be expected, so that the inhibition of the extrusion machinery has no consequences. In any case, since the experiments were based on the measurement of the accumulation not of an antibiotic but of AO, it could indicate that reflux is negligible in these bacteria.

Dendrimer **1** caused a marked increase in AO accumulation in *E. coli* 208691, suggesting that the efflux machinery was inhibited (Figure 5). However, this effect was not concentration-dependent at the concentrations tested, as no clear differences were observed between the MIC, ½ MIC or ¼ MIC, thereby suggesting that the maximum achievable inhibition occurs at concentrations below ¼ MIC; this would be consistent in determining synergism with antibiotics to which resistance depends on the efflux. AO accumulation at different concentrations of dendrimer 2 was similar to that observed by the inhibitor PaβN, whereas dendrimer 3 led to an increase in the AO accumulation with clear differences between the MIC, ½ MIC and ¼ MIC.

In *P. aeruginosa* ATCC 27853, a slight increase in AO accumulation was observed in the presence of the dendrimers 1 and 3 at MIC, whereas all three dendrimers inhibited efflux in a concentration-dependent manner, reaching higher inhibition than that caused by the inhibitor PaβN in the ciprofloxacin-resistant clinical isolate *P. aeruginosa* 666 SJD. These observations open new perspectives on the use of peptides for antimicrobial purposes, since resistance to many antibiotics is strictly due to efflux, while in others such as fluoroquinolones, the contribution of the efflux machinery is required to express resistance genes [35]. In fact, the search for efflux inhibitors is a prominent field of research.

It has been pointed out that some EPIs may be much more active on a specific pump; for instance, there are EPIs that specifically inhibit MexAB-OprM among all resistance–nodulation–division (RND) family pumps of *P. aeruginosa* [36,37]. If this is so, differences in inhibition should be expected depending on the substrate. To confirm the eventual interest of the dendrimers as EPIs, the interaction between the dendrimers 1 and 2 and small antibiotics such as rifampicin and ciprofloxacin was explored; in both cases, resistance has been attributed totally or partially to efflux. In MDR *E. coli*, both dendrimer 1 and dendrimer 2 were synergistic with ciprofloxacin (fractional inhibitory concentrations index, FICI, of 0.27 and 0.49, respectively), while in susceptible strains, an additive effect was seen. On the other hand, in *P. aeruginosa*, values of FICI revealed an additive effect in both susceptible and resistant strains. Results for rifampin were quite similar. It should be taken into account that efflux plays a major role in determining ciprofloxacin resistance in *E. coli* [38].

## 3. Conclusions

Peptides are an important class of organic molecules with broad biological activity and diverse chemical structures. Cyclic and branched peptides are examples of how the topology of a molecule contributes to biological activity. Here, we have demonstrated that 1,3,5-triazine (TA), through its trifunctional precursor, 2,4,6-trichloro-1,3,5-triazine (TCT), is an excellent branching unit for peptides. The different reactivities of the three Cl atoms present in the TCT allow the substitution of the first Cl by the N-terminal function of a peptide and then the two remaining Cl atoms could be easily functionalized with ethylenediamine (EDA) for the continuation of two identical peptide chains. Our synthetic strategy is fully compatible with the Fmoc/tBu SPPS methodology. We were able to fine-tune the condition for reacting TCT with the α-amino function of a peptide on resin at 0 °C without cross-linking. The two remaining Cl atoms were then replaced by two ethylendiamine (EDA) molecules under mild conditions, 45 °C for 3 h; in this case, again, the substitution was quantitative and no cross-linking was observed. The simplicity of this strategy circumvents the need to use p-hydroxybenzoic acid (PHBA) as an enhancer linker between the peptide chain and the TA, because the phenol was demonstrated to be a good leaving against the nucleophilic amine of the incoming EDA, favoring the loss of the TA moiety. Therefore, generations 1 and 2 of the (KL)_3_ peptide were efficiently prepared. Generation 1 (**2**) has three copies of the peptide, while generation 2 (**3**) has five copies. This work exemplified the strength of the solid-phase synthesis methodology for the preparation of peptides. It allows rapid preparation of peptides presenting diverse building blocks for the screening of their properties and if necessary, it is easily up-scalable.

Regarding the antimicrobial activity, our results show selectivity towards Gram-negative organisms, and the bacterial strains were susceptible only to the full dendrimers (**1**–**3**), while the hexapeptide (**A**) or the truncated dendrimers (**C**–**E**) were not active. Additionally, the presence of the TA moiety on the construct has been revealed as key for the antimicrobial activity, since the dendrimer of generation 1 containing Lys instead of the TA (**F**, mimic of **2**), and the 18-amino-acid linear peptide containing nine repeats of LK (**G**) did not show significant antimicrobial activity against the strains tested.

Dendrimers **1**, **2** and **3** did not induce membrane channels but caused drastic morphology alterations of the bacterial surface, as determined by AFM. The three molecules enhance the AO accumulation and interact with efflux machinery antibiotic extrusion, and subsequently should be regarded as hypothetical EPIs that deserve further research along these lines.

Furthermore, dendrimers **1** and **2** have remarkable antimicrobial activity against *E. coli* and *P. aeruginosa* MDR isolates, especially dendrimer **2**. Additionally, they were synergic with ciprofloxacin and rifampicin.

Given that dendrimer **2** is relatively easy to synthesize, lacks problematic side reactions, shows good antimicrobial activity and interferes with efflux, coupled with the absence of hemolysis and toxicity in eukaryotic cells, it could be considered a lead for further development and a good candidate for further research.

## 4. Experimental Section

All reagents and solvents for the chemical syntheses were obtained from commercial suppliers and were used without further purification unless stated otherwise. Analytical HPLC was performed on an Agilent 1100 system using Chemstation software Version B.04.03 for data processing. Phenomenex C18 column (3 μm, 4.6 × 150 mm) was used with a flow rate of 1.0 mL/min, 30 °C column oven and UV detection at 220 nm. Buffer A, 0.1% TFA in H_2_O, and buffer B, 0.1% TFA in CH_3_CN, were used in the HPLC. A gradient elution of 5–95% in 15 min was considered for all peptides/dendrimers unless otherwise specified. LC-MS was performed on an Ultimate™ 3000, AerisTM 3.6 µm wide pore column, Phenomenex C18 (150 × 4.6 mm) column. Buffer A: 0.1% formic acid in H_2_O; buffer B: 0.1% formic acid in CH_3_CN.

### 4.1. Bacterial Strains and Reagents

*E. coli* ATCC 25922, *P. aeruginosa* ATCC 27853, *S. aureus* ATCC 29213, *P. aeruginosa* 666 SJD (ciprofloxacin-resistant), *P. aeruginosa* P12, P13, P15, P16 and P17 (MDR isolates) and *E. coli* 7987, 5545, 8248, 13974, 7259 and 208691 (MDR isolates) from our own collection were included in the present study.

Tryptone soy agar and acridine orange were purchased from Sharlau (Barcelona, Spain), and Mueller Hinton II broth cation adjusted (MHBCA) from Becton Dickinson Diagnostic Systems, Inc. (Sparks, MD, USA). Imipenem monohydrate and PaβN were purchased from Sigma-Aldrich Chemicals (Madrid, Spain). The live/dead kit solution was obtained from Invitrogen (LIVE⁄DEAD^®^ BacLightTM kit L7012, Eugene, OR, USA).

### 4.2. Peptide Synthesis

All peptides were synthesized at 0.1 mmol scale following a Fmoc/tBu strategy by means of DIC and Oxyma as coupling reagents on Fmoc-Rink-Amide AM resin (loading = 0.64 mmol/g) as solid support. Compounds **F** (H-[(LK)_3_]_2_-K-(LK)_3_-NH_2_) and **G** (H-(LK)_9_-NH_2_) were synthesized under microwave conditions using CEM Liberty Blue using the standard cycle provided by the manufacturer. The peptide dendrimers **1** to **3** and **A** to **E** were synthesized manually in polypropylene syringes fitted with polypropylene frits at room temperature. Fmoc was removed by treatment of the resin with a solution of 20% piperidine in DMF for 1 min and then repeated for another 7 min. The couplings were executed with a 3-fold excess (6-fold excess after branching) of Fmoc-amino acid, DIC and OxymaPure in a 1:1:1 ratio in DMF for 45 min.

#### 4.2.1. Incorporation of p-Hydroxybenzoic Acid (PHBA)

The coupling of PHBA was performed under standard conditions by using 3 equiv. of PHBA/DIC/Oxyma for 1 h at rt. However, after completion of the reaction, the peptidyl-resin was treated with 20% piperidine in DMF to cleave any possible ester formed during the coupling process.

#### 4.2.2. Incorporation of 2,4,6-Trichloro-1,3,5-triazine (TCT)

Next, 3 equiv. of TCT and DIEA dissolved in DCM were added to the PHBA-peptidyl-resin and kept reacting for 60 min at −20 °C to prevent double incorporation of the phenol group. In the case of peptidyl-resin, the reaction was carried out at 0 °C, in which a single amine group was incorporated.

The substitution of the other two Cl on the triazine moiety was achieved by adding 50 equiv. of EDA:DIEA (1:1) in DMF the presence for 12 h at rt in the case of PHBA containing peptides or 3 h at 45 °C when not.

Peptides **C**, **D** and **E** were acetylated by reaction with 10 eq. of acetic anhydride: DIEA (1:2) in DMF for 30 min.

After completion of the peptide elongation, the peptidyl-resin was washed with methanol and then dried under vacuum. Peptides were cleaved from the resin by treatment with TFA/H2O/TIS (triisopropylsilane) (95:2.5:2.5 *v*/*v*) for 1 h at room temperature. They were then precipitated by chilled diethyl ether, centrifuged, and the filtrate was then discarded. The solid peptide was washed twice with chilled diethyl ether and centrifuged. The filtrate was then discarded and the peptide dried overnight in a desiccator under vacuum. Peptides were dissolved in Millipore water and analyzed by RP-HPLC and LC-MS, after which they were purified by semi-prep HPLC to ≥95% homogeneity (Figures S2–S21).

### 4.3. Hemolysis Assay on Red Blood Cells (RBCs)

The hemolytic properties of the peptides were studied by determining the release of hemoglobin from sheep red blood cells (SRBCs), following a previously reported method with some modifications [39]. Whole sheep blood was obtained from United Scientific, SA. The SRBCs were centrifuged at 2000 rpm for 10 min to separate the plasma and obtain erythrocytes. The latter were washed 3 times using sterile PBS buffer until a clear supernatant was achieved. The washed SRBCs were resuspended in PBS buffer to give approximately 20% hematocrit and were then stored at 4 °C and used within 6 h. Twofold serial dilutions of the tested peptides, containing 180 µL of the tested peptides in PBS buffer, were prepared in 1.5 mL Eppendorf tubes. Next, 20 µL of the SRBCs was added to each Eppendorf tube and incubated at 37 °C for 30 min. The samples were then spun at 3000 rpm for 4 min at 4 °C in a Centurion Scientific K1015 Micro Prime Centrifuge. The supernatants were then transferred to a fresh 96-well microtiter plate, and the absorbance was measured at 405 nm in a SpectraMax M2 microplate reader. PBS was used as the negative control (0% hemolysis) and distilled water was used as the positive control (100% hemolysis). The percentage of hemolysis was then calculated using the formula below:

$$\%\;Hemolysis=\left(\frac{ABS-{ABS}_{0}}{{ABS}_{100}-{ABS}_{0}}\right)\times 100$$

where *ABS*_100_ and *ABS*_0_ are the absorbances of the samples at 100% and 0% hemolysis, respectively. The experiment was performed in triplicate (Figure S22).

### 4.4. Cytotoxicity

The cytotoxicity assay was performed in the human hepatocellular carcinoma cell line Hep G2 ATCC and murine L-929 fibroblasts (NCTC clone 929, ECACC 88102702) obtained from the laboratory of Dr. Pérez-Tomás (Cancer Cell Biology, University of Barcelona). The cytotoxicity of the selected peptides was determined by measuring the intracellular reduction of resazurin sodium salt (Sigma-Aldrich, St. Louis, MO, USA). Briefly, HepG2 and L-929 cells were grown in RPMI 1640 or MEM medium, respectively (Biochrom AG, Berlin, Germany), supplemented with 10% fetal bovine serum. Cells from pre-confluent cultures were harvested with trypsin-EDTA and maintained at 37 °C in 5% CO_2_. Then, 100 µL of each cell line was seeded into 96-well flat-bottomed microplates and incubated for 24 h at 37 °C. The medium was then replaced with 200 μL of medium containing the dendrimers at concentrations ranging from 64 to 0.125 μg/mL and the microplates were incubated for 24 h at 37 °C. Next, 20 μL of resazurin was added to each well and the plates were incubated under the same conditions for 24 h. Fluorescence was measured using a multiwell scanning spectrophotometer at an excitation wavelength of 530 nm and an emission wavelength of 590 nm. Cytotoxicity was then calculated by comparing the fluorescence of the treated and untreated cells (Figure S22).

### 4.5. Minimum Inhibitory Concentration (MIC)

The antimicrobial activity of the dendrimers was evaluated against *E. coli* ATCC 25922, *P. aeruginosa* ATCC 27853 and *S. aureus* ATCC 29213 strains. The MIC values were determined by the broth microdilution method. Briefly, the different strains were grown in Muller Hinton broth cation adjusted (MHBCA) medium overnight at 37 °C with shaking at 200 rpm. The bacterial cultures were then adjusted to OD_625nm_ of 0.08–0.1, and 5 μL of each diluted suspension was added to 96-well plates previously filled with MHBCA and serially diluted peptides. Plates were incubated for 24 h at 37 °C. Finally, the MIC was determined macroscopically, based on the visual turbidity of the wells.

### 4.6. Bacterial Growth Curves

The real-time effect of the peptide dendrimers was examined on the growth curves of *E. coli* ATCC 25922 and *P. aeruginosa* ATCC 27853. Volumes of 10 mL of TSB liquid cultures containing 1–5 × 10^5^ CFU/mL in the logarithmic phase were adjusted in Falcon tubes. Peptide dendrimers were then added at MIC and sub-inhibitory concentrations (½ MIC and ¼ MIC). Incubation was performed in RTS-1C real-time reverse spin bioreactors RTS-1 (Biosan) for 24 h at 37 °C and 2000 rpm. Growth was measured noninvasively as optical density (OD 850 nm) every 15 min.

### 4.7. Time-Kill Curves

The death kinetics of the active dendrimers were examined by plotting their time-kill curves against *E. coli* ATCC 25922 and *P. aeruginosa* ATCC 27853. The dendrimers were added to a starting bacterial inoculum of 10^6^ CFU/mL in 5 mL of MHBCA medium. Dendrimers were tested at three different concentrations: MIC, ½ MIC and ¼ MIC. They were incubated for 24 h at 37 °C with shaking at 200 rpm. Samples were aseptically collected at 0, 1, 2, 4 and 6 h, serially diluted in Ringer ¼ and plated on Tryptone soy agar for colony counting.

The response of the microbial strains to the dendrimers was determined based on a logarithmic decrease in viable bacteria. A compound was considered active if it caused a reduction of ≥1 log_10_ CFU/mL, compared to the corresponding counts at time 0 h. In addition, a dendrimer was considered bactericidal if it caused a reduction of ≥3 log_10_ CFU/mL [40].

### 4.8. Black Lipid Bilayers

The basic methods have been described previously [41]. Membranes were prepared from 1% lipid a mixture of di-phytanoyl-phosphatidylcholine/di-phytanoylphosphatidylserine (DiPhPC/PS) in a molar ratio of 4:1 in n-decane (Avanti Polar Lipids, Delfzyl, The Netherlands). Bilayers were painted over a 2 mm^2^ hole in a Teflon divider separating two compartments containing 5 mL each of a bathing solution of 1MKCl. Voltages were applied across the membrane via calomel electrodes connected by a salt bridge. The resultant current was amplified 10^8^-fold by a current amplifier and recorded on a Rikadenski chart recorder. A concentration of 1 μg/mL of the dendrimer was used based on the results of Wu et al. [42].

### 4.9. Atomic Force Microscopy

*E. coli* ATCC 25922 was grown at 37 °C in Mueller Hinton cation-adjusted broth to an optical density of 0.25 at 625 nm. Dendrimers were added to a starting inoculum of 10^6^ CFU/mL and incubated for 4 h at 37 °C with shaking. Untreated *E. coli* was included as a control. Cells in 1 mL of culture were centrifuged at 5000 rpm for 5 min and washed three times with 1 mL of sterile water. The bacterial cells were then resuspended in 100 µL of sterile water and a 10 µL drop was applied to a freshly cleaved mica surface and allowed to dry for approximately 10 min at room temperature.

The disk surfaces were analyzed by atomic force microscopy using an AFM XE-70 (Park Systems, Suwon, Korea) in non-contact mode with an ACTA 10M cantilever. The rectangular-shaped cantilever had a force constant of 40 N/m, a resonance frequency of 300 kHz and a tip radius with a curvature of <10 nm. The AFM images were processed using a scanning probe image processor XEI (Park Systems), correcting the plane of the AFM image for any coupling of the lateral plane and the *z*-axis caused by the instrument [43,44].

### 4.10. Intracellular Accumulation of Acridine Orange

The assay was performed as previously described by Armengol et al. [45] including control and clinical strains (*E. coli* 208691 and *P. aeruginosa* 666 SJD) previously studied by our group [45]. Briefly, 96-well flat-bottomed microtiter plates (Guangzhou Jet Bio-Filtration Co., Ltd., Mianyang, China) were used. Overnight cultures were diluted in 1/4 Ringer’s solution to an OD_520nm_ of 1.5, and 100 µL was added to the wells previously filled with 100 µL of Ringer’s solution. Acridine Orange (AO) was added to a final concentration of 0.25 µg/mL. The inoculated plates were then incubated for 30 min with shaking, after which the fluorescence was measured in a FLUOstar OPTIMA fluorescence microplate reader (BMG Labtech, Ortenberg, Germany).

To determine the uptake and accumulation of AO by bacteria, different scenarios were compared. First, bacteria were examined in the presence of PAβN at 20 µg/mL, and then in the presence of different concentrations of the dendrimers at (MIC, ½ MIC, ¼ MIC). The results in each condition were expressed as the percentage increase in fluorescence with respect to the control. To ensure that the conditions used in the AO accumulation experiments did not affect the viability of the bacterial cells, live-dead determinations of cell viability were performed.

### 4.11. Confocal Laser Scanning Microscopy (CLSM)

Briefly, bacterial suspensions of the different strains studied with an OD_520nm_ of 1.5 in Ringer’s solution were placed on the eight-well glass plates. They were treated with 0.25 µg/mL AO and dendrimers. The inoculated plates were then incubated for 30 min at 37 °C, after which the staining mixture (1.5 µL of SYTO 9 and 1.5 µL of propidium iodide in 1 mL of Ringer ¼) (LIVE/DEAD ^®^, BacLightTM, Viability Kit L7012) was added and incubated for another 15 min more in the dark. Fluorescence was examined by CLSM using a Leica TCS-SL filter-free spectral confocal laser-scanning microscope (Leica Microsystems, Mannheim, Germany) equipped with a 488 nm argon laser, 543 nm and 633 nm He/Ne lasers, and a 63× magnification oil immersion objective (Scientific and Technological Centers, University of Barcelona, L’Hospitalet de Llobregat, Spain). CLSM images were analyzed using Image J software 2.1.0/1.53c (National Institutes of Health, Bethesda, MD, USA).

### 4.12. Checkerboard Assay

A checkerboard assay was used to determine the fractional inhibitory concentrations (FICs) of dendrimers in combination with conventional antibiotics. Briefly, each well in a 96-well plate was inoculated with 100 µL of a bacterial inoculum of 1 × 10^6^ CFU/mL, and the plates were incubated at 37 °C for 24 h. The FIC was calculated after identifying the first well in each row without growth (MIC), according to the following formula: FIC of drug A (FIC A) = (MIC of drug A in combination)/(MIC of A); FIC of drug B (FIC B) = (MIC of drug B in combination)/(MIC of B). The FIC index (FICI) values were calculated by adding the FIC of drug A to the FIC of drug B [46]. The interpretation of the (FICI) was the following: FICI ≤ 0.5, synergy; 0.5 < FICI ≤ 1, additivity; 1 < FICI ≤ 2 antagonism [47].

## Figures and Tables

**Table 1:** MIC (μg/mL) for new dendrimer/peptide constructs.

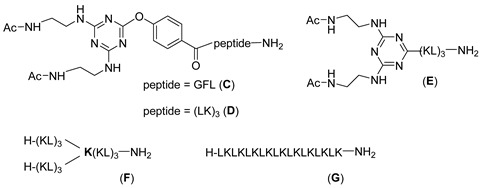				
Dendrimer	Sequence	MIC (μg/mL)		
		*S. aureus* ATCC 29213	*E. coli* ATCC 25922	*P. aeruginosa* ATCC 27853
**1**	H-[(LK)_3_]_2_-(EDA)_2_-TA-PHBA-(LK)_3_-NH_2_	>64	8	16
**2**	H-[(LK)_3_]_2_-(EDA)_2_-TA-(LK)_3_-NH_2_	>64	2	16
**3**	H-[(LK)_3_]_4_-(EDA)_4_-(TA)_2_-(EDA)_2_-TA-(LK)_3_-NH_2_	>64	8	32
**A**	H-(LK)_3_-NH2	>64	>64	>64
**B**	H-[(LK)_3_]_2_-(EDA)_2_-TA-PHBA-GFL-NH_2_	>64	64	32
**C**	(Ac-EDA)_2_-TA-PHBA-GFL-NH_2_	>64	>64	>64
**D**	(Ac-EDA)_2_-TA-PHBA-(LK)_3_-NH_2_	>64	>64	>64
**E**	(Ac-EDA)_2_-TA-(LK)_3_-NH_2_	>64	>64	>64
**F**	H-[(LK)_3_]_2_-K-(LK)_3_-NH_2_	64	32	>64
**G**	H-(LK)_9_-NH_2_	>64	>64	>64

MICs (minimal inhibitory concentrations) were determined as the lowest concentrations of the peptides that prevent visible bacterial growth. ATCC = American Type Culture Collection.

**Table 2:** Susceptibility to dendrimeric constructs, ciprofloxacin and colistin of MDR isolates belonging to *E. coli* and *P. aeruginosa*.

	MIC (μg/mL)			
Isolate	Dendrimer 1	Dendrimer 2	Ciprofloxacin	Colistin
*E. coli* 7987	8	4	32	ND
*E. coli* 5545	8	8	64	ND
*E. coli* 8248	8	8	32	ND
*E. coli* 13974	8	8	32	ND
*E. coli* 7259	32	2	32	ND
*E. coli* 208691	32	8	256	0.5
*P. aeruginosa* P12	>128	32	64	64
*P. aeruginosa* P13	64	32	128	32
*P. aeruginosa* P15	>128	16	256	>128
*P. aeruginosa* P16	64	32	64	16
*P. aeruginosa* P17	16	16	256	>8

## Data Availability

The original contributions presented in the study are included in the article/Supplementary Material, further inquiries can be directed to the corresponding author.

## Supplementary Materials

The following supporting information can be downloaded at: https://www.mdpi.com/article/10.3390/ijms25115883/s1.
