# Supplementary material for: 1,3,5-Triazine as Branching Connector for the Construction of Novel Antimicrobial Peptide Dendrimers: Synthesis and Biological Characterization
Source: Int J Mol Sci. 2024 May 28;25(11):5883. doi: 10.3390/ijms25115883 (PMC11172478; doi:10.3390/ijms25115883)
Supplement: Supplementary file 1 [file ijms-25-05883-s001.zip › ijms-3004957-supplementary.pdf]

Supplementary Information (SI)

1,3,5-Triazine as Branching Connector for the Construction of Novel Antimicrobial Peptide Dendrimers. Synthesis and Biological Characterization

Rotimi Sheyi, Jessica T. Mhlongo, Marta Jorba, Ester Fusté, Anamika Sharma, Miguel Viñas, Fernando Albericio, Paula Espinal, and Beatriz G. de la Torre

<u>Table of content</u>

Truncated dendrimer structures…………………………..…………………………S1

HPLC and Mass Spectra ………………………………………..…………..S2-S21

Hemolysis and cytotoxicity……………………………………………………..S22

Confocal light microscopy ……………………………………………………..S23

Figure S1: Truncated dendrimers found during the synthesis of G2 dendrimer containing PHBA.

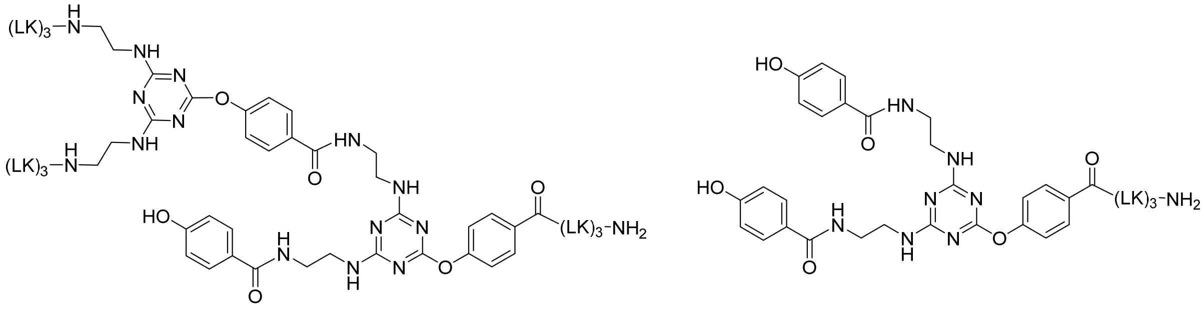

Figure S2: HPLC trace of purified **1** (H-[(LK)$_3$]$_2$-(EDA)$_2$-TA-PHBA-(LK)$_3$-NH$_2$).
Method: 15-50% B into A in 15 min

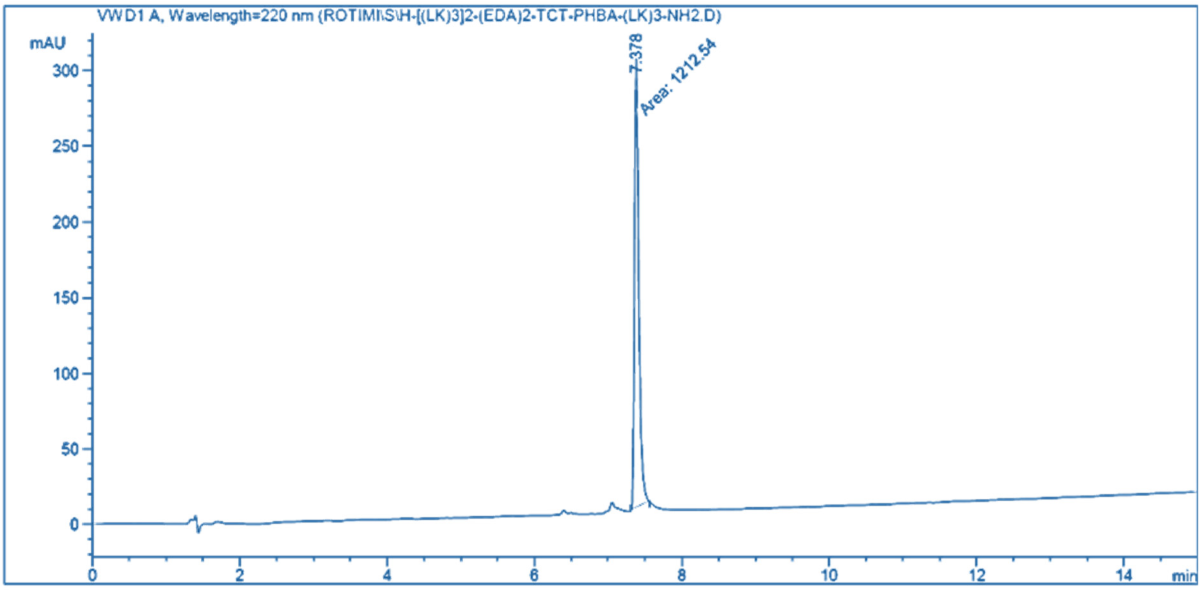

Figure S3: ESI-MS of **1** (H-[(LK)$_3$]$_2$-(EDA)$_2$-TA-PHBA-(LK)$_3$-NH$_2$).
Expected mass= 2,505.37
Observed mass = (627.21 $[M+4H]^+/4$, 501.81 $[M+5H]^+/5$, 418.57 $[M+6H]^+/6$)

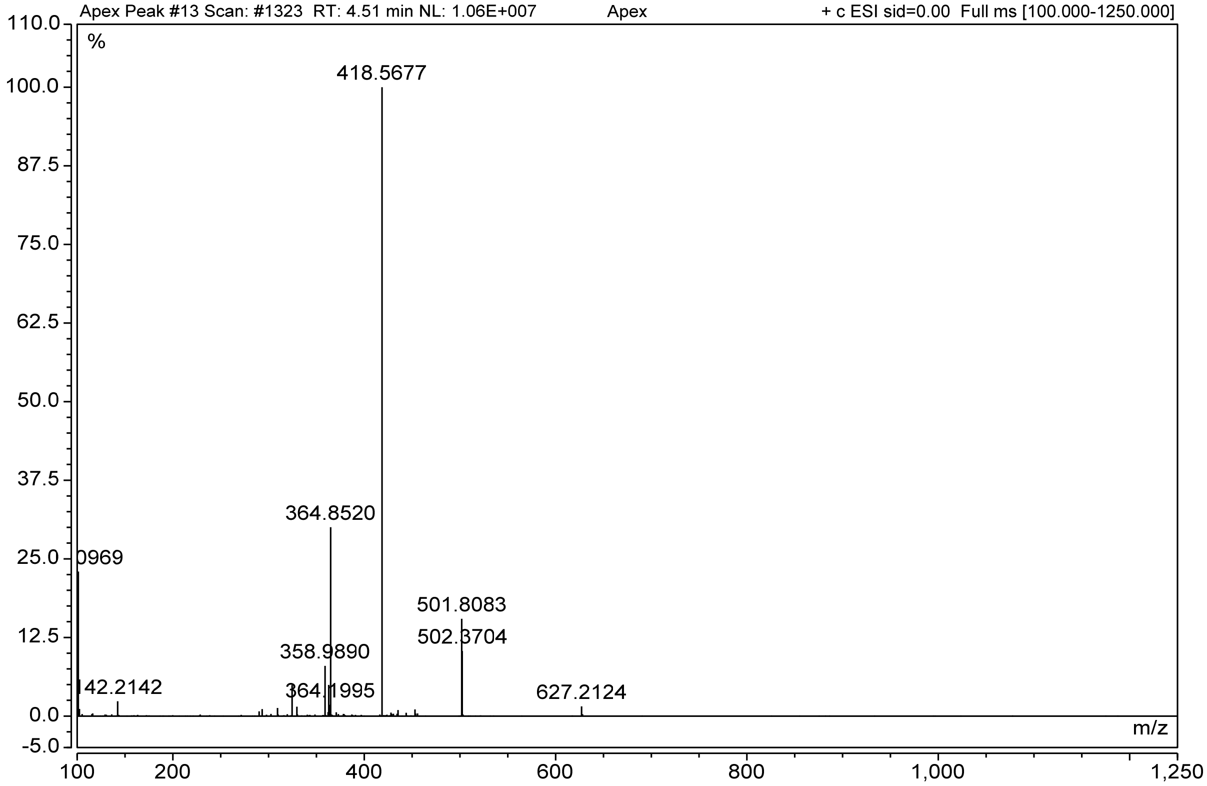

Figure S4: HPLC trace of purified **2** (H-[(LK)$_3$]$_2$-(EDA)$_2$-TA-(LK)$_3$-NH$_2$).
Method 15-50% B into A in 15 min

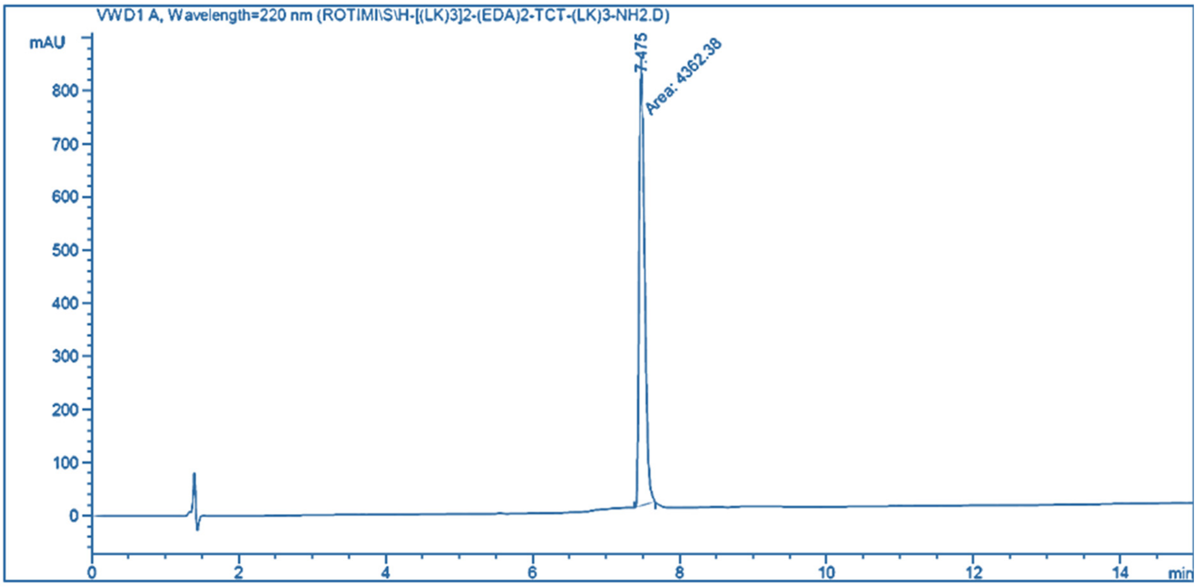

Figure S5: ESI-MS of **2**: (H-[$(LK)_3]_2$-$(EDA)_2$-TA-$(LK)_3$-NH_2)
Expected mass= 2385.26
Observed mass = (597.27 $[M+4H]^+/4$, 478.07 $[M+5H]^+/5$, 398.41 $[M+6H]^+/6$)

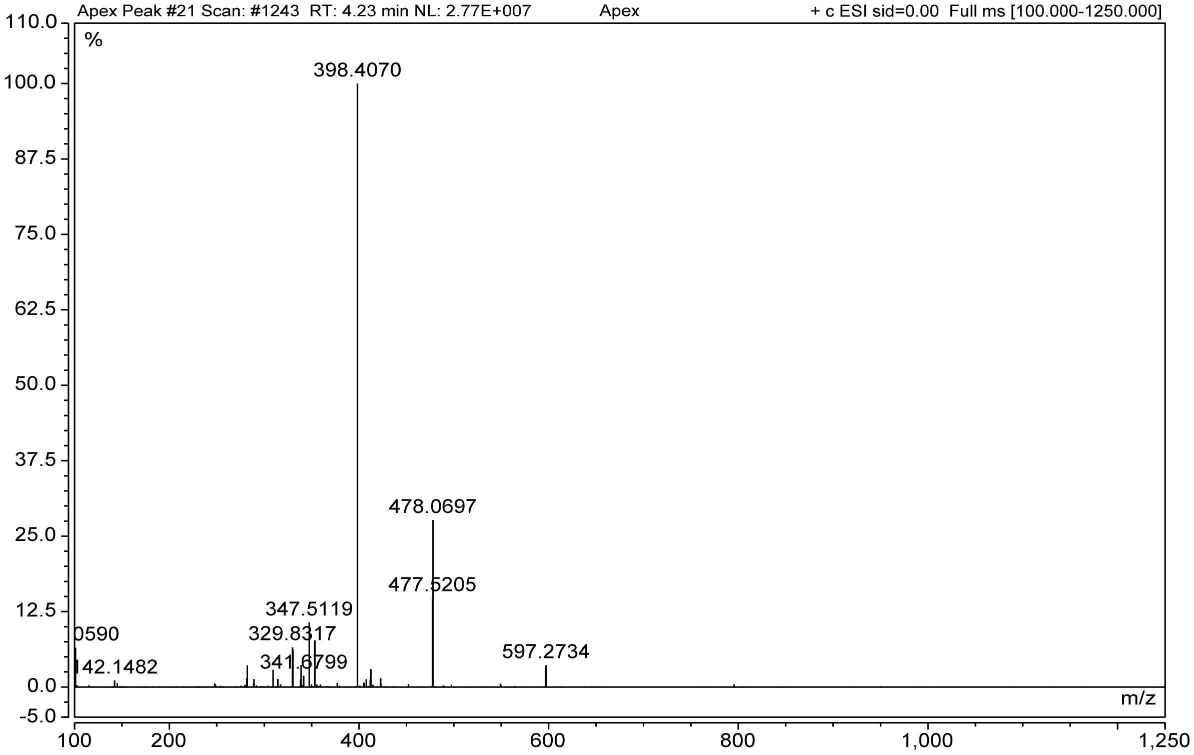

Figure S6: HPLC trace of purified **3** (H-[$(LK)_3]_4$-$(EDA)_4$-$(TA)_2$-$(EDA)_2$-TA-$(LK)_3$-NH_2).
Method 20-40% B into A in 15 min

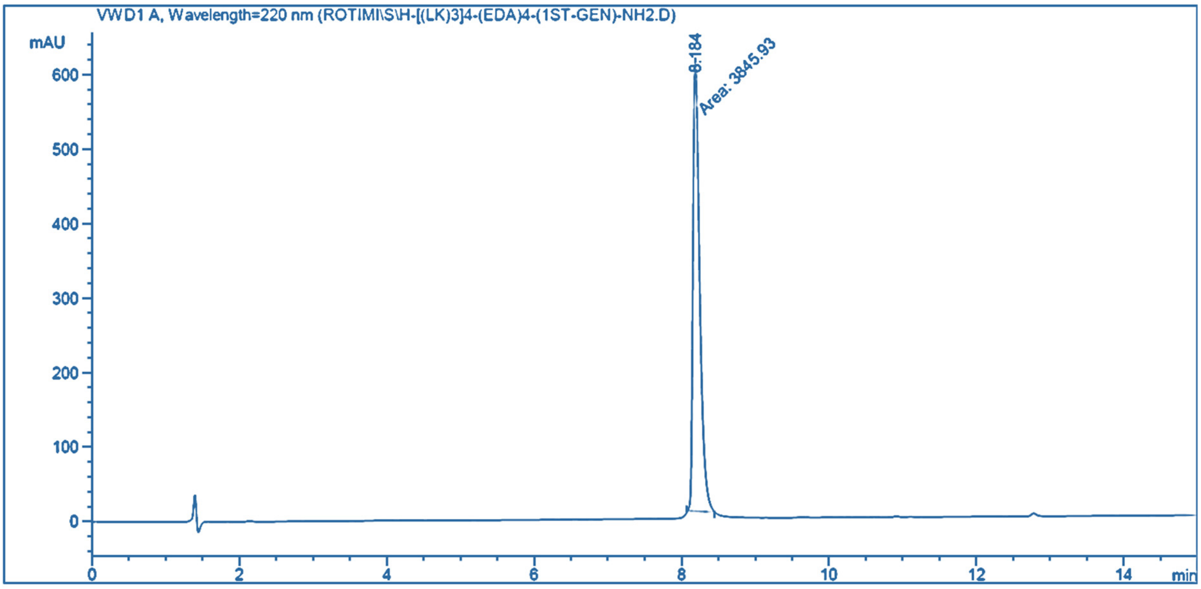

Figure S7: ESI-MS of **3**: (H-[$(LK)_3]_4$-$(EDA)_4$-$(TCT)_2$-$(EDA)_2$-TCT-$(LK)_3$-NH_2).
Expected mass= 4223.73
Observed mass = (705.04 [M+6H] +/6, 604.42 [M+7H] +/7, 528.99 [M+8H] +/8)

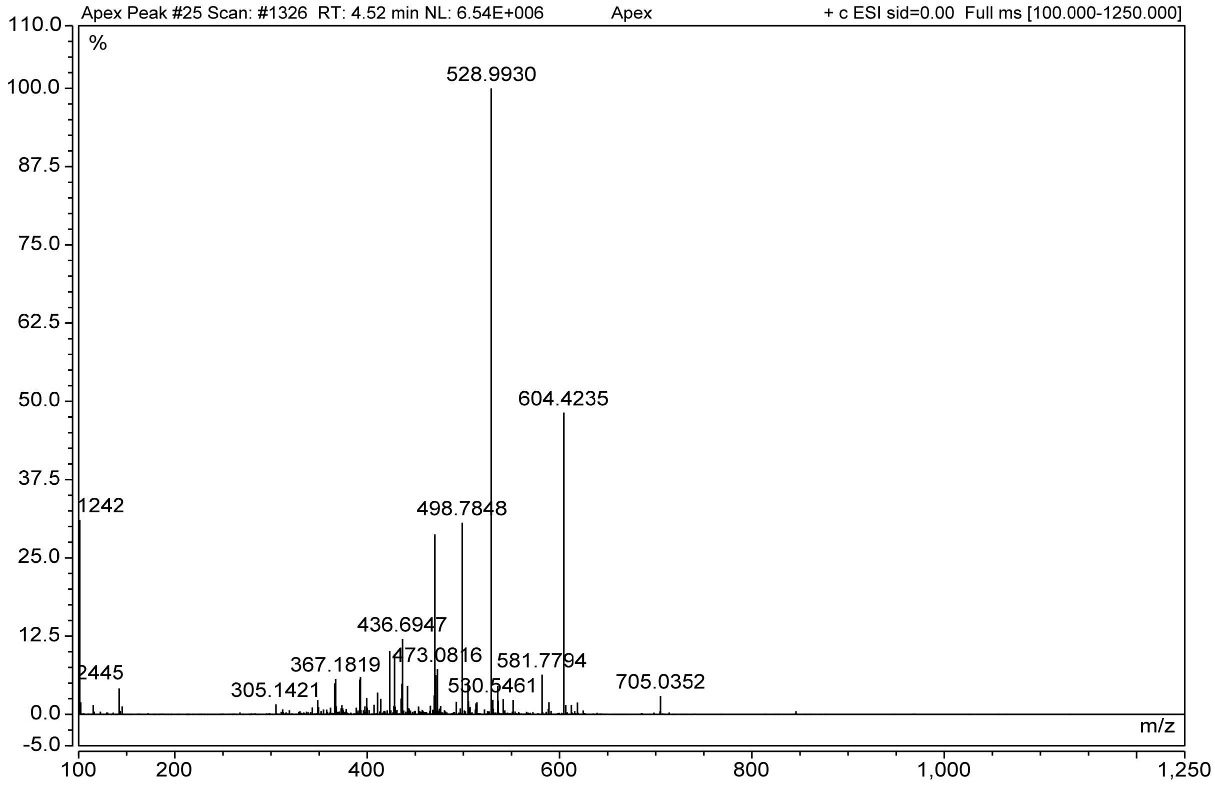

Figure S8: HPLC trace of purified **A** (H-$(LK)_3$-NH_2).
Method 5-95% B into A in 15 min

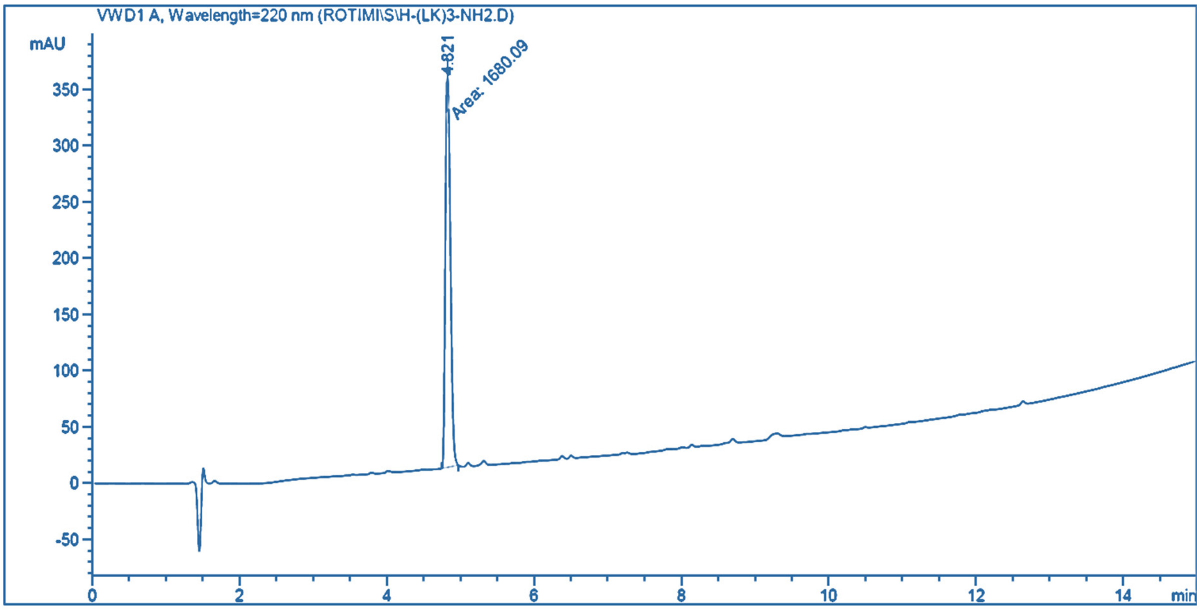

Figure S9: ESI-MS of **A**: (H-$(LK)_3$-NH_2).
Expected mass= 741.04
Observed mass = (741.62 $[M+H]^+$, 371.44 $[M+2H]^+/2$, 248.24 $[M+3H]^+/3$)

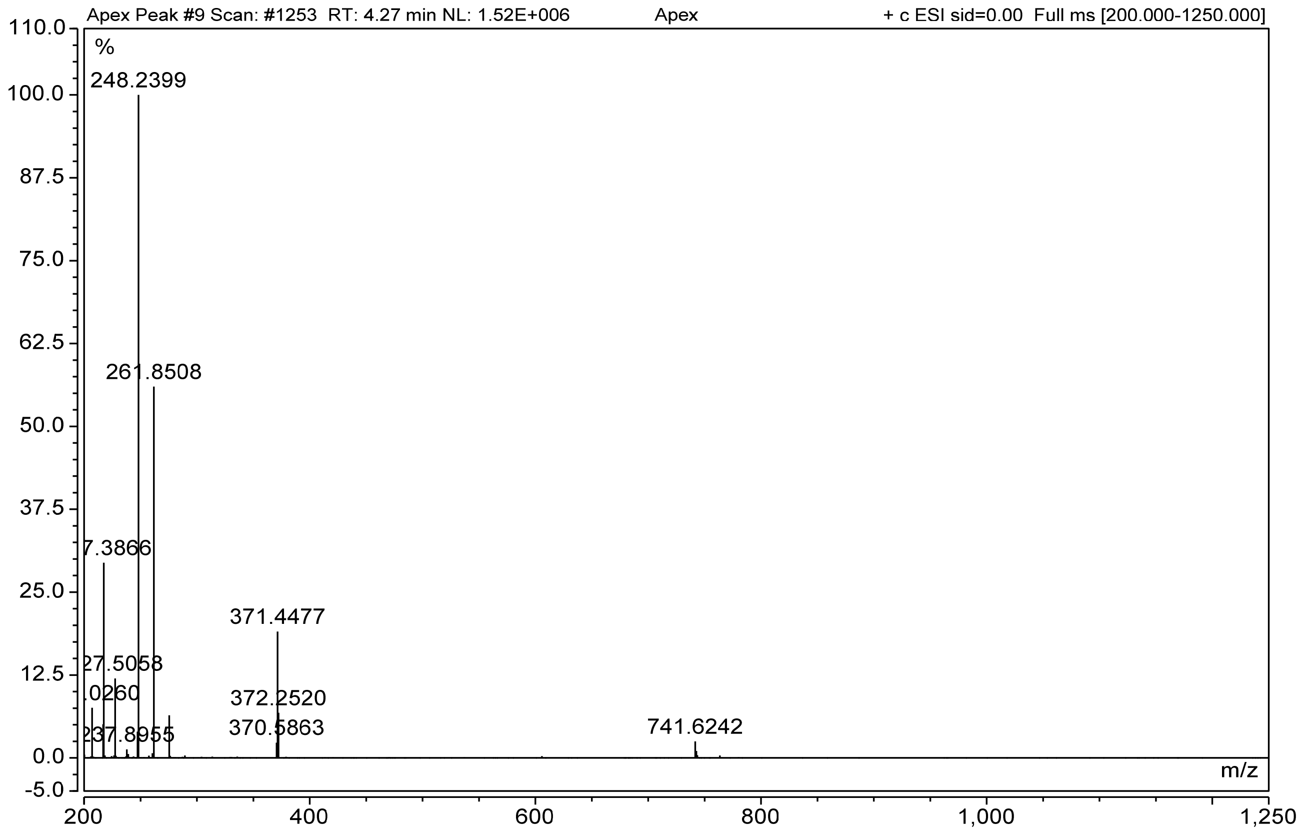

Figure S10: HPLC trace of purified **B** (H-$[(LK)_3]_2$-$(EDA)_2$-TA-PHBA-GFL-NH_2).
Method 15-70% B into A in 15 min

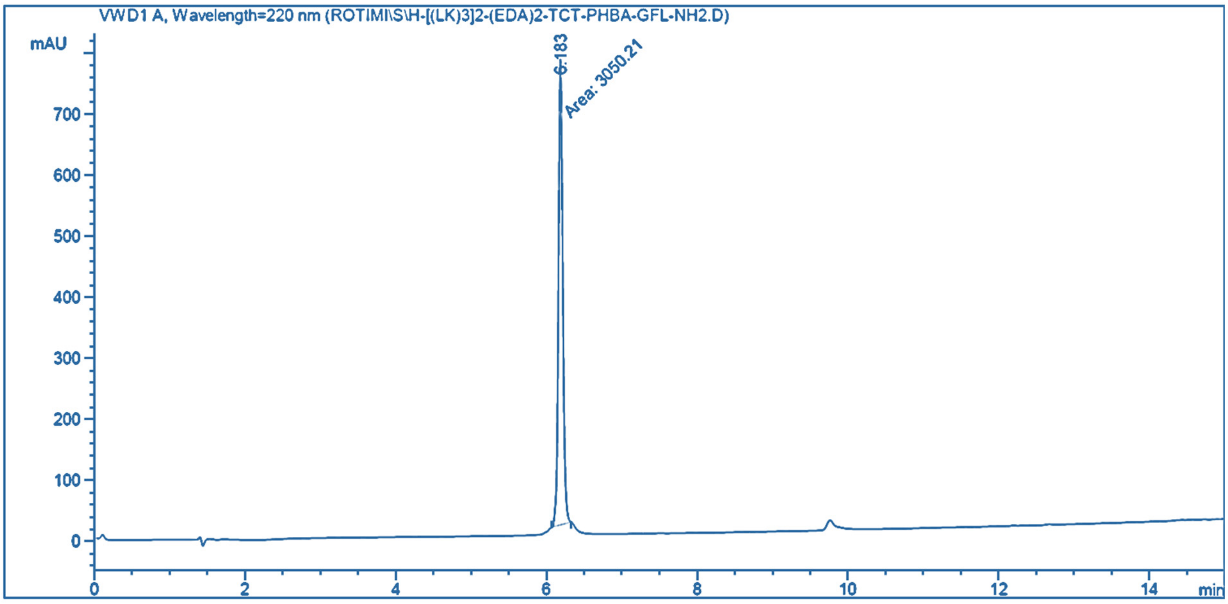

Figure S11: ESI-MS of **B**: (H-[(LK)$_3$]$_2$-(EDA)$_2$-TA-PHBA-GFL-NH$_2$).
Expected mass= 2097.77
Observed mass = (700.14 [M+3H]$^+$/3, 525.57 [M+4H]$^+$/4)

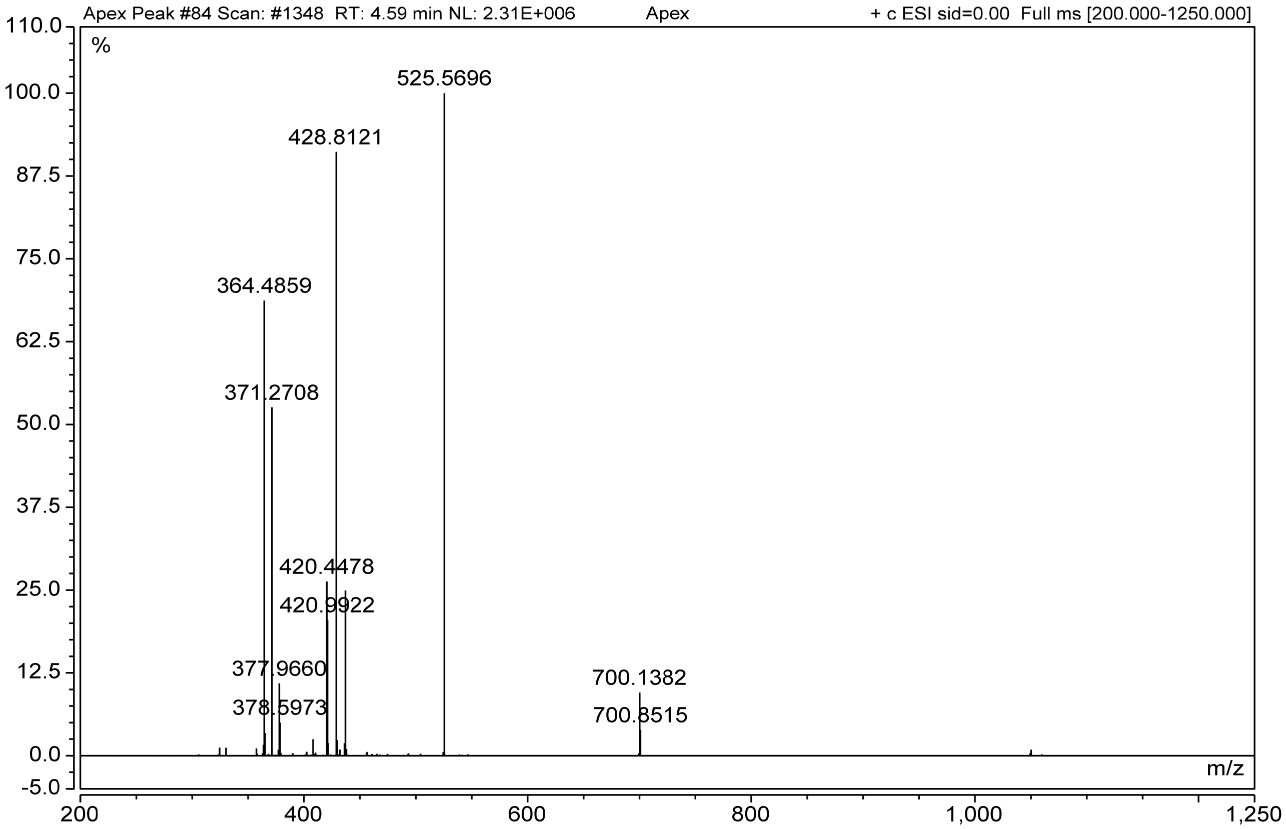

Figure S12: HPLC trace of purified **C** ((Ac-EDA)$_2$-TA-PHBA-GFL-NH$_2$).
Method 15-40% B into A in 15 min

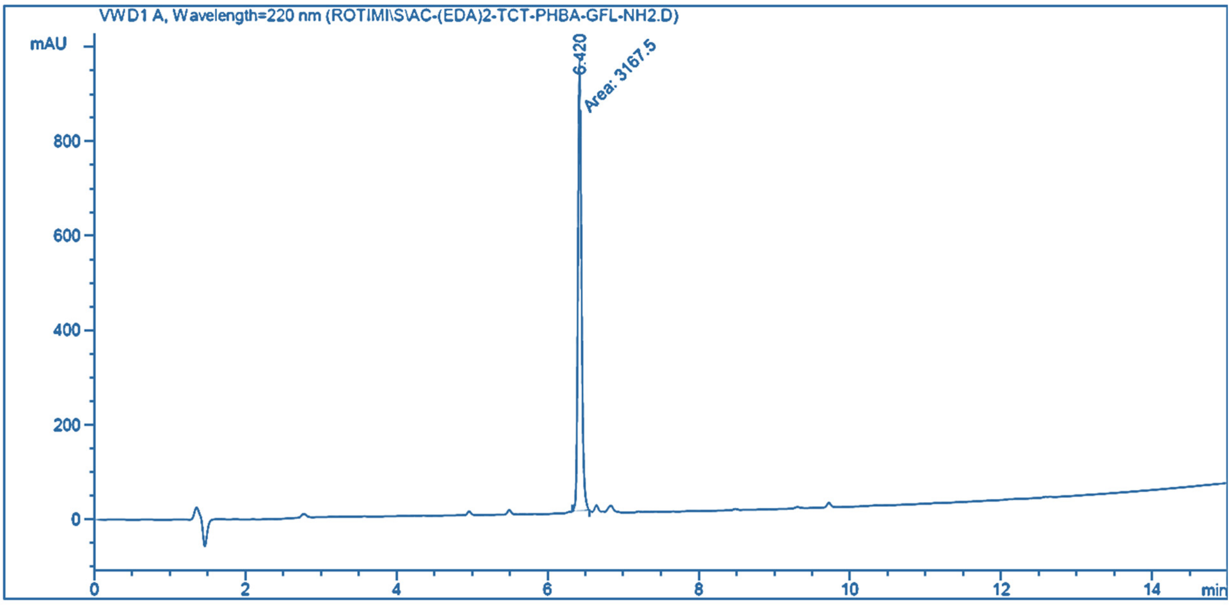

Figure S13: ESI-MS of **C**: ((Ac-EDA)$_2$-TA-PHBA-GFL-NH$_2$).
Expected mass= 733.83
Observed mass = (734.30 [M+H]$^+$, 368.07 [M+2H]$^+$/2)

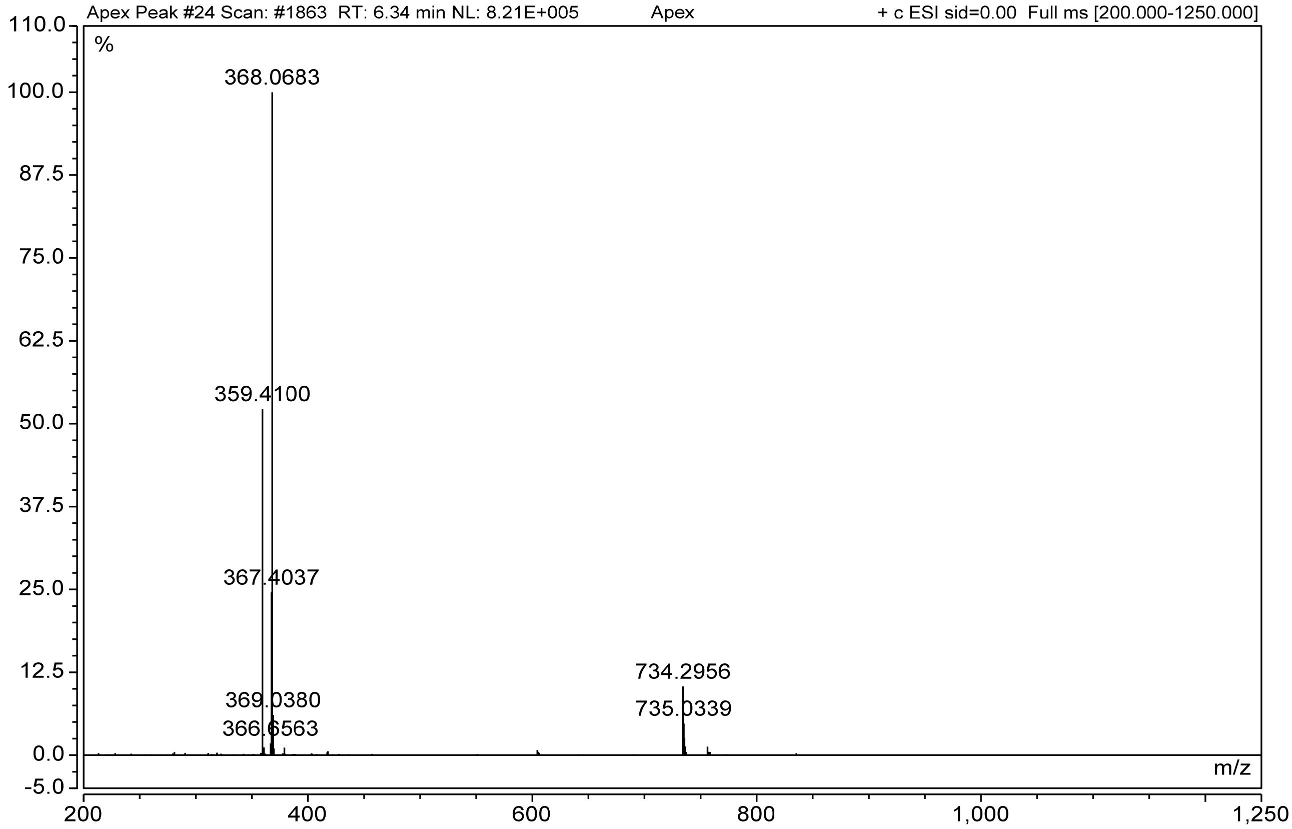

Figure S14: HPLC trace of purified **D** ((Ac-EDA)$_2$-TA-PHBA-(LK)$_3$-NH$_2$).
Method 15-50% B into A in 15 min

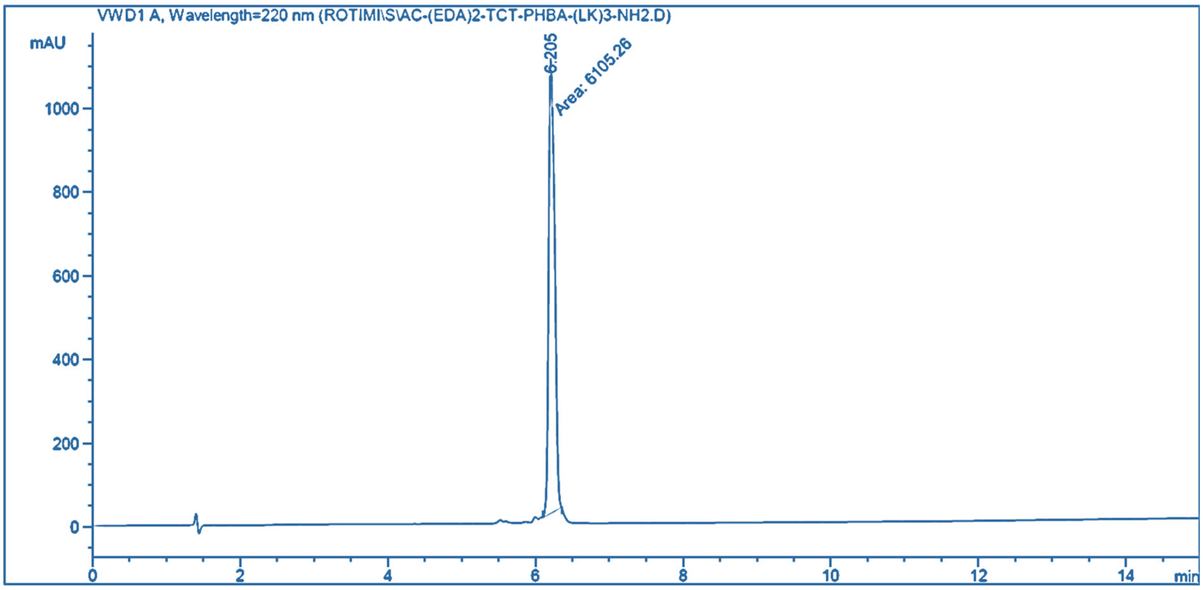

Figure S15: ESI-MS of **D**: ((Ac-EDA)$_2$-TA-PHBA-(LK)$_3$-NH$_2$).
Expected mass= 1141.43
Observed mass = (570.91 [M+2H]$^+$/2, 381.19 [M+3H]$^+$/3, 286.14 [M+4H]$^+$/4)

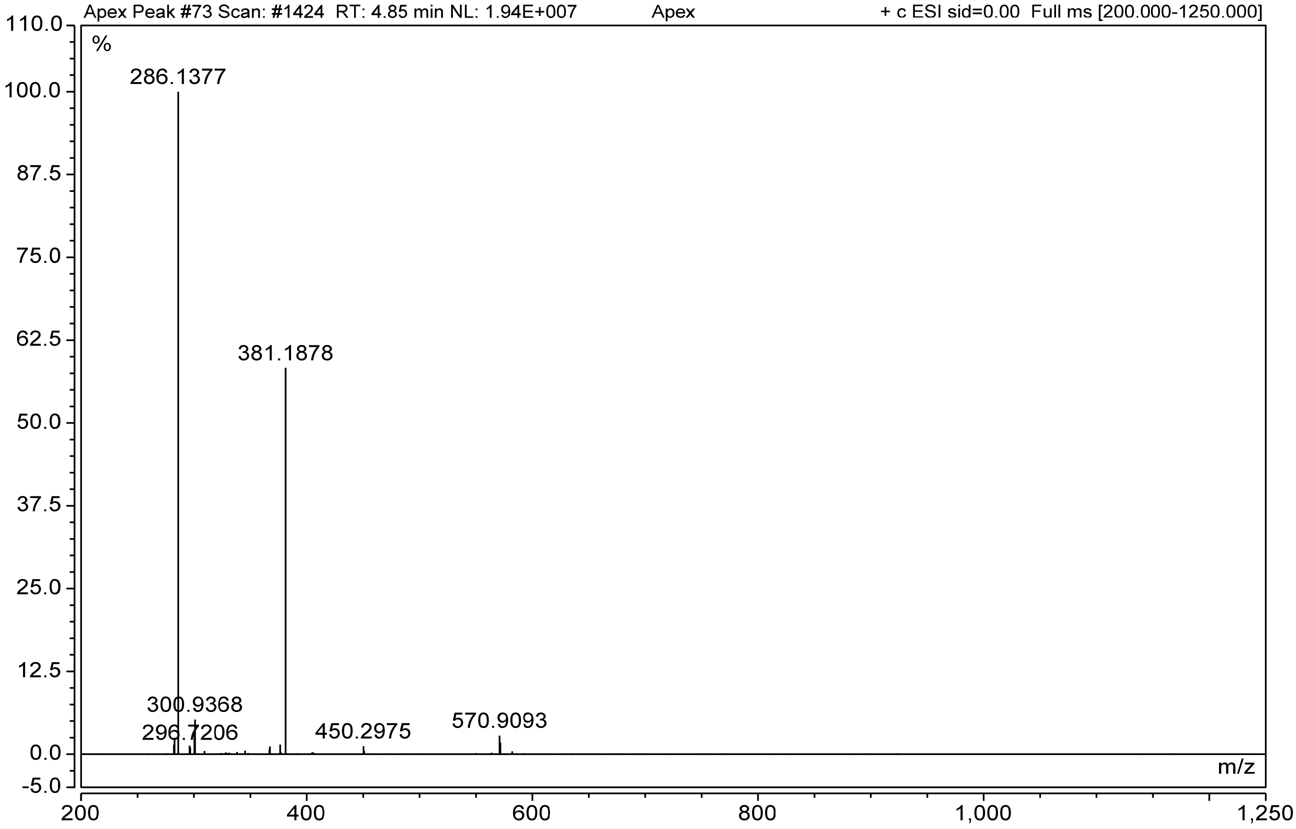

Figure S16: HPLC trace of purified **E** ((Ac-EDA)$_2$-TA-(LK)$_3$-NH$_2$).
Method 15-50% B into A in 15 min

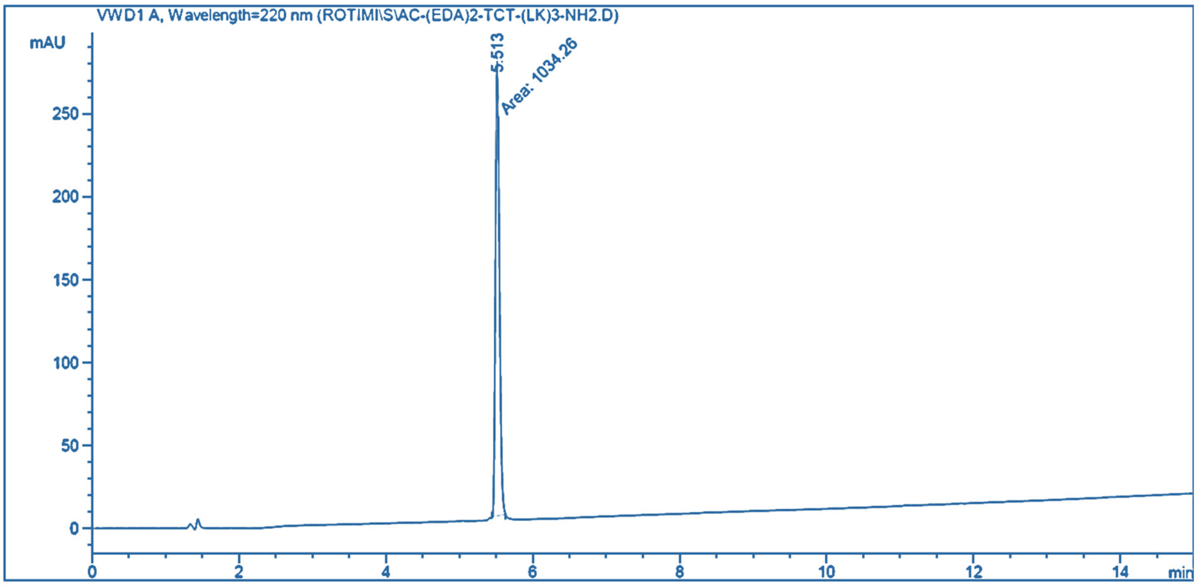

Figure S17: ESI-MS of **E**: ((Ac-EDA)$_2$-TA-(LK)$_3$-NH$_2$).
Expected mass= 1021.32
Observed mass = (511.30 [M+2H] +/2, 341.11 [M+3H] +/3, 256.12 [M+4H] +/4)

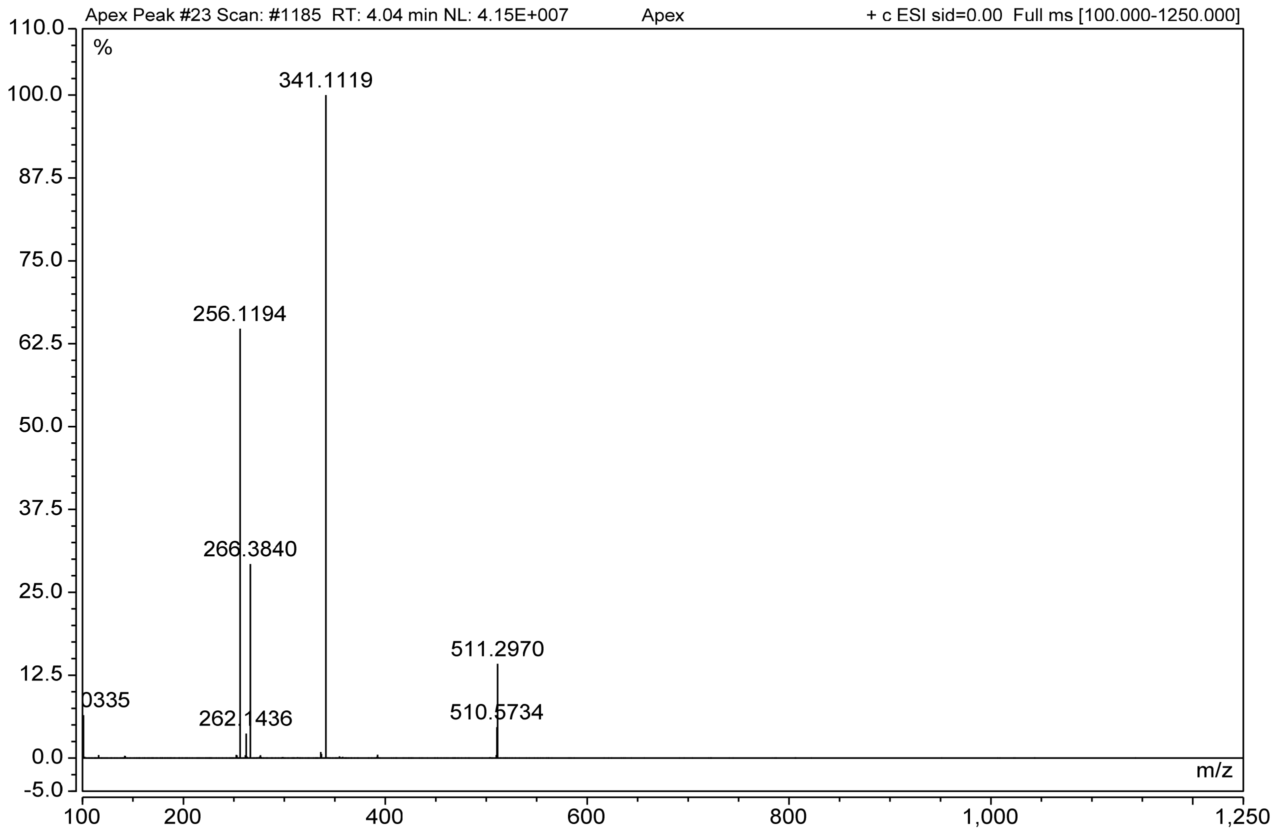

Figure S18: HPLC trace of purified **F** (H-[(LK)$_3$]$_2$-K-(LK)$_3$-NH$_2$).
Method 15-50% B into A in 15 min

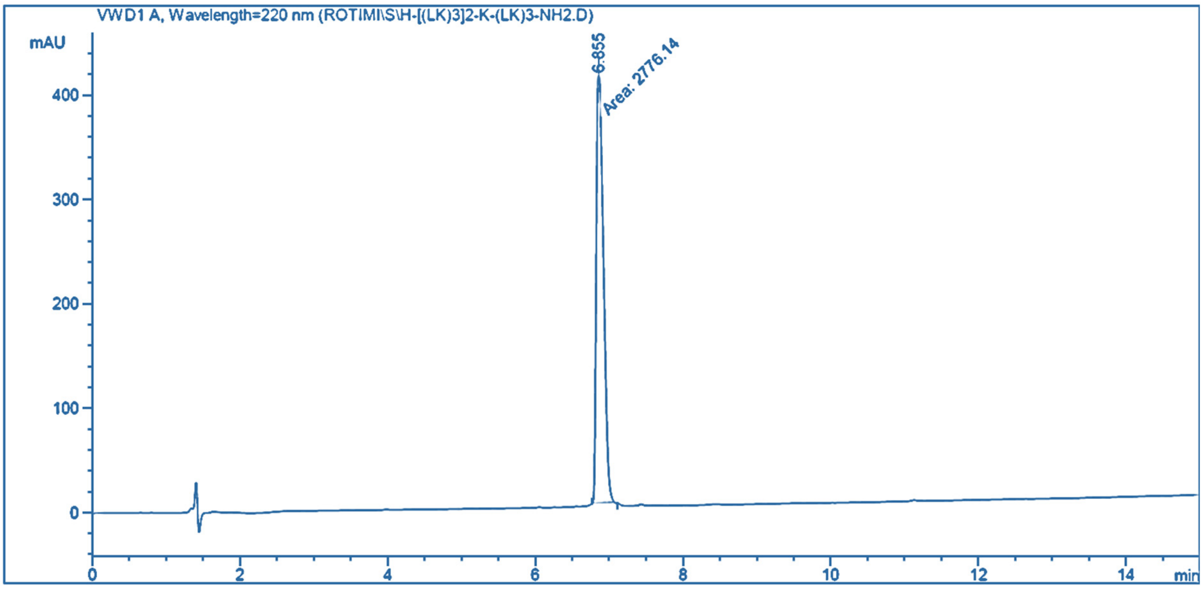

Figure S19: ESI-MS of **F**: (H-[(LK)$_3$]$_2$-K-(LK)$_3$-NH$_2$).
Expected mass= 2317.22
Observed mass = (580.38 [M+4H]$^+$/4, 464.49 [M+5H]$^+$/5, 387.24 [M+6H]$^+$/6)

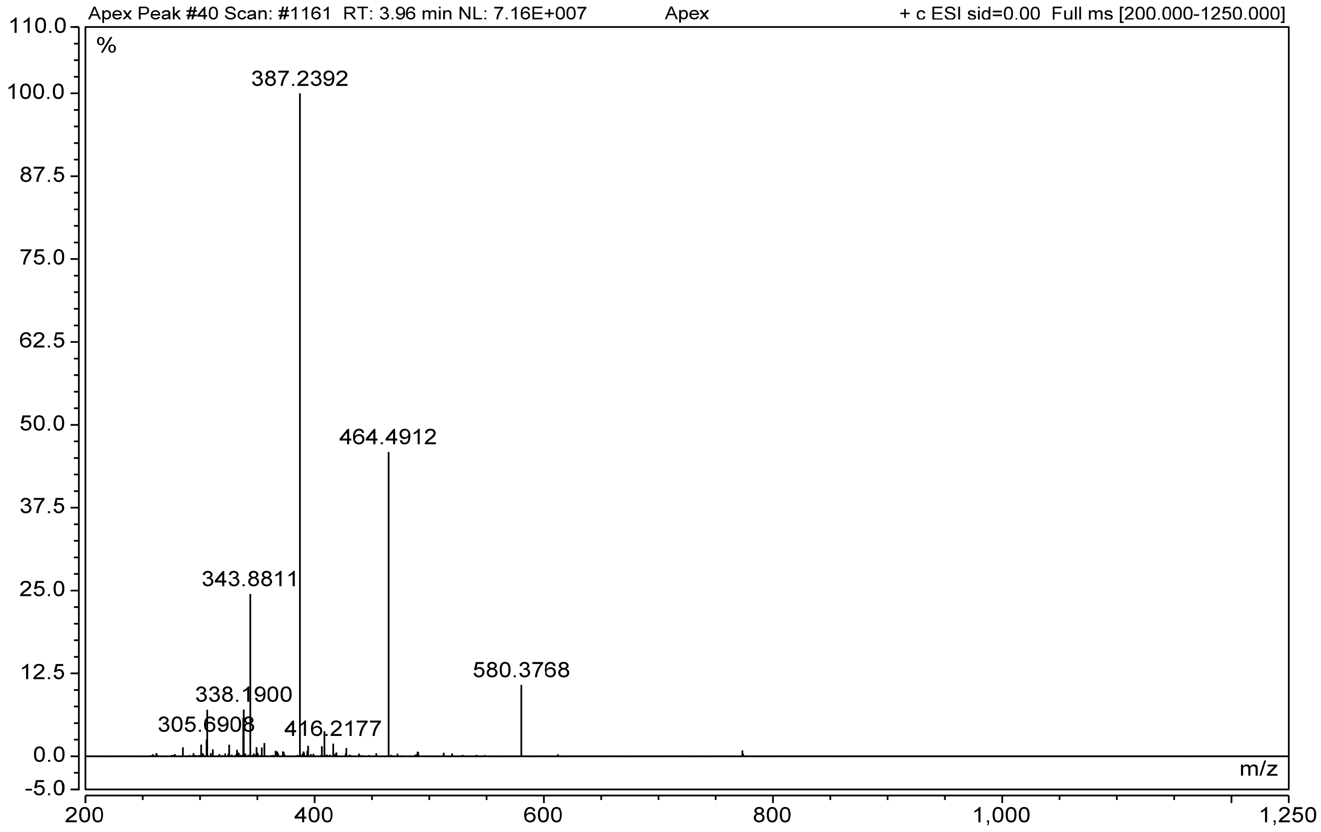

Figure S20: HPLC trace of purified **G** (H-(LK)$_9$-NH$_2$).
Method 15-50% B into A in 15 min

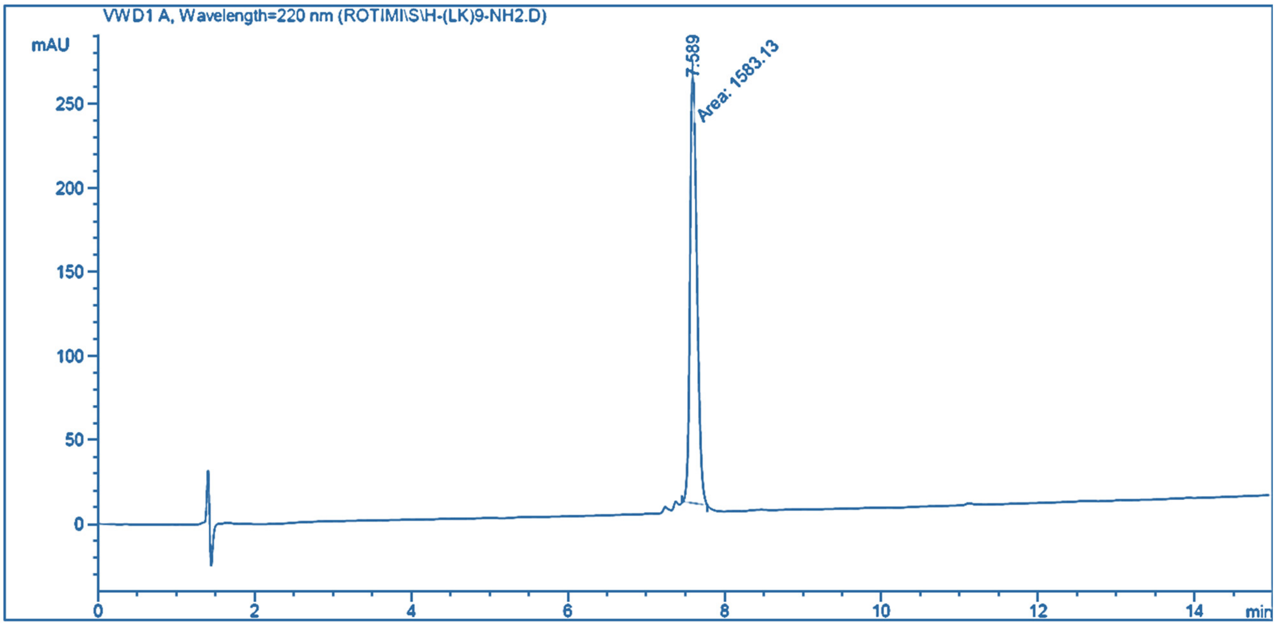

Figure S21: ESI-MS of **G**: (H-(LK)9-NH2).
Expected mass= 2189.05
Observed mass = (730.53 [M+3H] +/3, 548.33 [M+4H] +/4, 438.87 [M+5H] +/5 365.90 [M+6H] +/6)

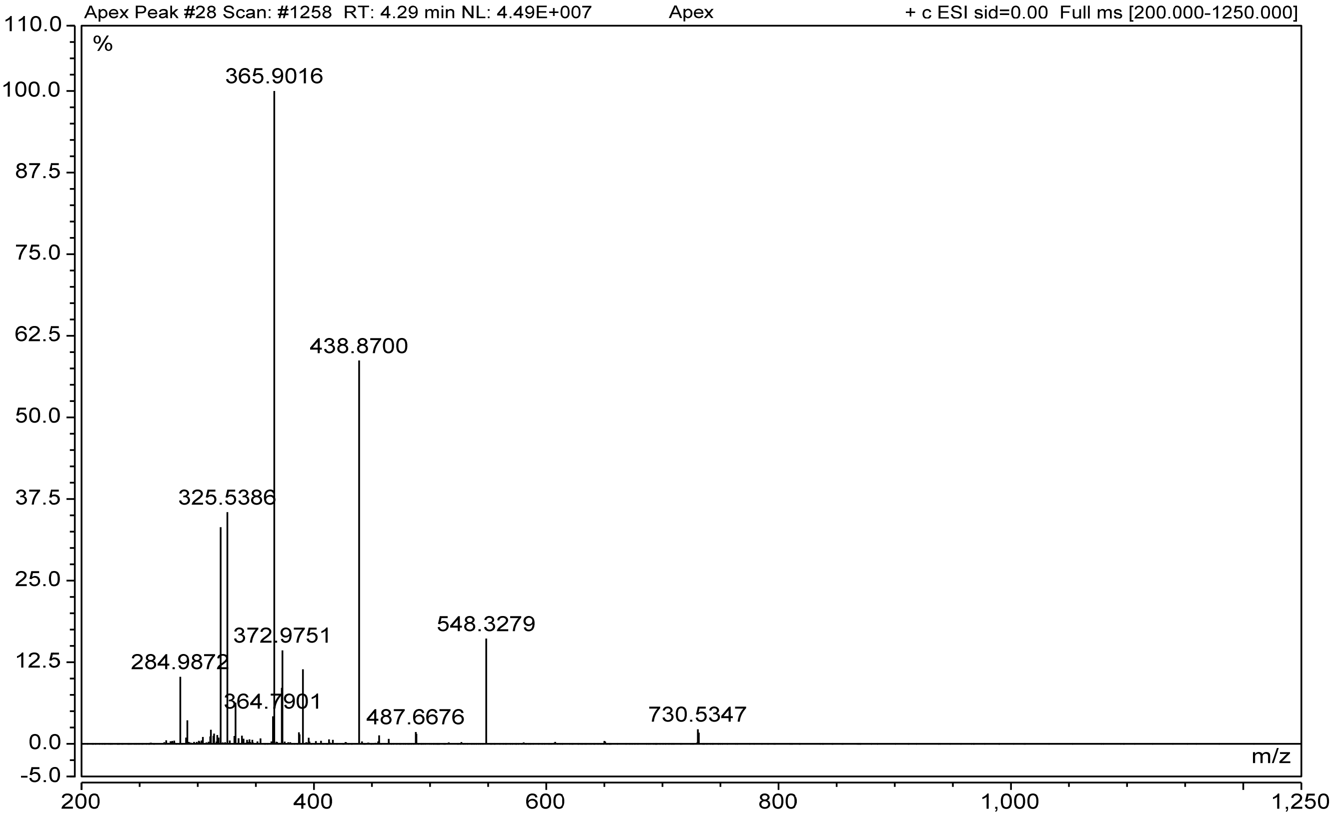

Figure S22: Hemolysis and cytotoxicity at 64µg/mL of dendrimers 1,2, and 3.

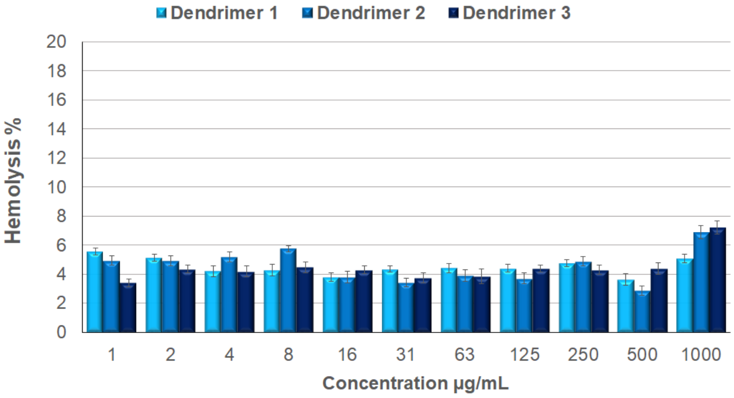

% Cytotoxicity at 64µg/mL

	L-929	HepG2
**Dendrimer 1**	2.8	0
**Dendrimer 2**	0	0
**Dendrimer 3**	0	0

Figure S23: Confocal light microscopy imaging after Live/Dead staining of *E. coli* 208691.

(A) **(B)**

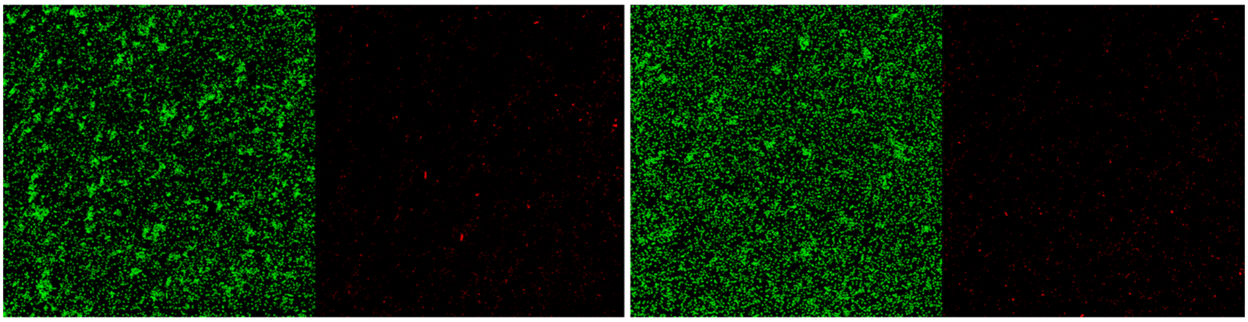

(A) Untreated cells (*E. coli)* ; (B) *E. coli* treated with 0.25 µg/mL of AO and dendrimer 2 at MIC. Similar images were obtained for all the isolates and dendrimers included in the intracellular accumulation of acridine orange assay.
